# NDRG3 is essential for sustaining antigen-driven T cell responses by protecting against restimulation-induced cell death

**DOI:** 10.1093/jimmun/vkag070

**Published:** 2026-05-14

**Authors:** Brian J Stack, Susan M Kaech

**Affiliations:** NOMIS Center for Immunobiology and Microbial Pathogenesis, Salk Institute for Biological Studies, La Jolla, CA, United States; Biological Sciences Graduate Program, University of California San Diego, La Jolla, CA, United States; NOMIS Center for Immunobiology and Microbial Pathogenesis, Salk Institute for Biological Studies, La Jolla, CA, United States

**Keywords:** T cells, NDRG3, restimulation-induced cell death

## Abstract

During the response to infections and pathogenic challenges, T cells must expand profoundly and be resilient to repetitive restimulation to clear the ongoing assault and protect the host. This process of prolific expansion is tightly regulated to quickly provide a robust pool of pathogen-fighting T cells, yet also limit excessive expansion to prevent inadvertent damage to host tissues. Restimulation-dependent pro-growth and pro-death signals help regulate this delicate balance of T cell expansion to maintain both host and T cell homeostasis. We have discovered that NDRG3 is a critical determinant of whether T cells proliferate or undergo apoptosis during repetitive restimulation under antigen-driven T cell expansion. CD8^+^ T cells lacking NDRG3 exhibit severely impaired expansion in vivo in response to both viral infections and tumor challenges. We show that NDRG3 is essential for T cell survival during antigen restimulation by protecting T cells from restimulation-induced cell death (RICD), while it has only a marginal impact on T cell survival in contexts with limited antigen restimulation. Mechanistically, NDRG3 safeguards repetitively stimulated T cells from RICD by constraining FAS-mediated pro-death signaling through caspase-8. Furthermore, NDRG3 overexpression enhanced T cell infiltration into tumors, improving their tumor-controlling capacity. Collectively, these findings establish NDRG3 as a novel, indispensable regulator of T cell responses to foreign challenges. Additionally, this work identifies NDRG3 as a previously undescribed regulator of RICD in T cells and reveals NDRG3 as a potential target for autoimmune disorders and chimeric antigen receptor T cell treatment for cancer.

## Introduction

T cells play an integral role in host protection by eliminating foreign pathogens and fighting cancer. To perform this essential function, naïve T cells must become activated by recognizing their cognate antigen (signal 1) with their T cell receptor (TCR) in the presence of a major histocompatibility complex, receive costimulatory signals through CD28 (signal 2), and engage inflammatory cytokine cues like IL-12 and IFN-α (signal 3).^1^^,^^2^ These signals induce a suite of signaling and transcriptional programs that activate T cell proliferation, glycolytic metabolism, effector cell differentiation, and the expression of proinflammatory cytokines, like IL-2.^1^^,^^3^^,^^4^ These cellular processes collectively enable the rapid clonal expansion of antigen-specific T cells, increasing their numbers by as much as 50,000-fold to fight the foreign challenge.^5^^,^^6^ Despite the strength of these programs in inducing T cell expansion, there are mechanisms present to carefully regulate the size and duration of the T cell response in order to protect the host from excessive expansion of effector T cells. Two of those pro-apoptotic programs that help constrain the T cell response are cytokine withdrawal-induced death (CWID) and restimulation-induced cell death (RICD).^7–10^ These programs are functionally active at different stages of the T cell response as CWID plays a role during the contraction phase, whereas RICD helps restrict the size of the T cell pool during the expansion phase.^11^ CWID can be induced by the reduction in IL-2 signaling that happens during the resolution of infection.^12^ This reduction in IL-2 signaling induces the expression of pro-apoptotic transcription factors BIM and PUMA in T cells, which depolarize mitochondria to release cytochrome C and induce apoptosis.^13^^,^^14^ However, it is important to note that CWID is not solely responsible for the culling of effector T cells during the resolution of the anti-pathogen T cell response, as different differentiation states of effector T cells have differential cytokine dependencies, for instance, IL-15 is important for sustained terminal effector T cell persistence.^15^ On the other hand, RICD occurs when already-activated T cells receive restimulation of their TCR in the presence of strong IL-2 signaling. This restimulation can induce apoptosis and functions to limit T cell expansion in an antigen-specific manner.^10^

RICD is heavily dependent on FAS activation in T cells.^11^ FAS and its ligand, FASLG, are both upregulated by TCR stimulation, which is one of the primary reasons for why FAS is so strongly implicated with RICD.^16^ Once FAS is activated, FADD (FAS-associated protein with death domain) and caspase-8 (CASP8) are recruited to the intracellular domain of FAS. CASP8 then undergoes proteolytic processing to become active so it can cleave executioner caspases, like CASP3, to induce apoptosis.^17^ The importance of FAS signaling in T cells is highlighted by early work on the *lpr* and *gld* mutations in mice.^18–21^ The *lpr* and *gld* mutations are inactivating mutations in FAS and FASLG, respectively, and mice that carry those mutations exhibit excessive lymphoproliferation and impaired T cell apoptosis. Subsequent mechanistic work exhibited that activated T cells are susceptible to apoptosis in vitro when restimulating their TCR, but this effect was abrogated upon blocking FAS-FASLG interactions.^22^ Additionally, it was shown that TCR and FAS coengagement is required to achieve the full pro-apoptotic effect of FAS activation, illustrating that the mechanisms governing FAS-mediated RICD still rely on TCR stimulation as an activating cue.^23^ Despite this, there are endogenous mechanisms present to combat the potency of FAS signaling as c-FLIP acts as a negative regulator of CASP8 cleavage and activation.^24^ The loss of c-FLIP compromises T cell expansion in response to bacterial infection through enhancing RICD, highlighting the importance of negatively regulating FAS signaling to prevent a reduction of the anti-pathogen T cell response.^25^ FAS is an important regulator of RICD and T cell survival, but further work is needed to fully understand the complex regulatory network that controls T cell survival during times of repeated antigen exposure.

In this work, we reveal that N-Myc downstream regulated gene 3 (NDRG3) plays a critical role in protecting T cells from RICD and is essential to sustain antiviral and antitumor T cell responses. There are 4 members of the NDRG family that share about 53% to 65% amino acid sequence identity with each other.^26^^,^^27^ They belong to the alpha/beta hydrolase superfamily of proteins that contain carboxypeptidases and lipases. However, none of the NDRG family members possess the amino acid residues necessary to form a functional catalytic triad required for hydrolytic activity.^28–31^ Despite this, they have been shown to act as influential scaffold proteins that have demonstrated roles in regulating a wide array of cellular functions ranging from cellular migration, to responding to cellular stress cues, to regulating cancer metastasis.^26^^,^^32–37^ NDRG3 protein has also been shown to be degraded in the absence of lactate in certain cell lines, indicating that there may be an added layer of regulation of NDRG3 at the protein level.^35^ Additionally, recent work from Komorowska et al.^38^ showed that NDRG3 was important for establishing the naïve T cell pool in mice. Given these interesting findings, we set out to understand how NDRG3 regulates T cell biology in the context of infection and disease and discovered that NDRG3 is absolutely necessary to sustain antiviral and antitumor T cell responses through restraining RICD-mediated elimination.

## Methods

### Mice

C57BL/6J were purchased from the Jackson Laboratory. Use of P14^+^ mice has been previously described.^39^ Use of *Rag1*^−/−^ mice has been previously described.^40^ Both male and female mice between 6 and 12 wk of age were used. In all experiments, age- and sex-matched mice were randomly assigned to experimental groups. In P14^+^ T cell cotransfer experiments, paired littermates were used as donors. Animals were housed in specific pathogen–free facilities at the Salk Institute with a cycle of 6:00 to 18:00 light and 18:00 to 6:00 dark, room temperatures from 20 to 22 °C, and a humidity range of 30% to 70%. All experimental studies were approved and performed in accordance with guidelines and regulations implemented by the Salk Institute Animal Care and Use Committee. The maximal tumor size permitted by our Institutional Animal Care and Use Committee of 2 cm^3^ for subcutaneous tumors were not exceeded during the period of observation.

### Infections

Mice were infected with 2 × 10^5^ plaque-forming units LCMV-Armstrong by intraperitoneal injection. Mice were infected with 2 × 10^6^ plaque-forming units LCMV-Clone 13 by retro-orbital injection. LCMV-Armstrong and LCMV-Clone 13 stocks were prepared as previously described.^41^

### Cell lines and cell culture

The B16-gp33 cell line was a gift from Hanspeter Pircher (University of Freiburg, Germany).^42^ B16-gp33 was cultured in Dulbecco’s Modified Eagle Medium with 10% fetal bovine serum (FBS), 1% penicillin-streptomycin, and 250 µg/mL G418 (Invitrogen; #10131027). HEK293T cells were cultured in Dulbecco’s Modified Eagle Medium with 10% FBS and 1% penicillin-streptomycin. Primary murine T cell culture media is referred to as T cell media and is made with RPMI 1640, 10% FBS, 1% penicillin-streptomycin, 2 mM L-glutamine, and 50 μM β-mercaptoethanol.

### Tumor implantation and regimen of tumor size quantification

B16-gp33 cells were dissociated from culture using 0.25% trypsin, washed, and resuspended in phosphate-buffered saline (PBS). Mice were anesthetized under continuous isoflurane, and one flank of the mouse was shaved to expose the injection site. The skin was then gently lifted using forceps, and 4 × 10^5^ cells were subcutaneously injected in a volume of 100 μL using an insulin syringe with a 29-gauge needle (BD). B16-gp33 was implanted 8 d before adoptive transfer of P14^+^ T cells. Tumor size was measured via calipers on the day of adoptive transfer of P14^+^ T cells and every 2 to 3 d post–tumor engraftment. Tumor volume was calculated with the following calculation: volume = (4/3) × (π) × (length/2) × (width/2) × (width/2).

### Cell isolation

Spleens were mechanically dissociated with 1 mL syringe plungers over a 70 µm nylon strainer. Spleens were incubated in ACK buffer for 3 min after dissociation over nylon strainer. B16-gp33 tumors were minced with scissors and incubated in RPMI with 2% FBS and 1 mg/mL collagenase IV, and 0.1 mg/mL DNase1 for 30 min at 37 °C with gentle shaking and then strained over 70 µm nylon strainers. Strained tumor samples were then incubated in ACK buffer for 3 min. Lysis was stopped with addition of 5 mL of T cell media.

### Flow cytometry and cell sorting

Cell suspensions were first incubated with eBioscience Fixable Viability Dye eFluor 780 (Viability Dye 780) for 5 min at room temperature. Cells were stained with primary surface antibodies (Table 1) in PBS with 2% FBS (flow buffer) for 35 min on ice. For sorting, staining was performed in PBS with 2% FBS, 2 mM EDTA, and 10 mM HEPES. For experiments requiring intracellular staining, cells were fixed with fixation buffer (BD) for 10 min at room temperature after primary surface antibody incubation. Cells were then incubated with eBioscience Foxp3/Transcription Factor Staining kit fixation/permeabilization buffer for 45 min on ice. Intracellular marker staining was performed in 1× permeabilization buffer for 45 min on ice. Flow cytometry analysis samples were acquired on a BD FACSymphony A3 or a BD Accuri C6. Cell sorting was performed on a BD FACSAria Fusion.

**Table 1:** Research material information.

Flow cytometry antibodies and dyes				
Target	Channel	Catalog number	Provider	Dilution
FAS	FITC	152606	BioLegend	200
FASLG	PE	12-5911-82	Thermo Fisher Scientific	200
BCL2	FITC	633504	BioLegend	200
Annexin V	APC	640920	BioLegend	20
Viability Dye 780	APC-Cy7	13–0865	Tonbo Biosciences	2,000
CD8a	BUV395	565968	BD Biosciences	400
CD44	BV711	103057	BioLegend	300
PD-1	BV785	135225	BioLegend	200
TIM-3	BV711	119727	BioLegend	200
SLAMF6	BV605	745250	BD Biosciences	200
Thy1.1	BUV737	741774	BD Biosciences	800
Thy1.2	BUV496	741046	BD Biosciences	400
Ly5.1	BV421	110732	BioLegend	200
Ly5.2	FITC	109806	BioLegend	200
Ly5.2	PE	109808	BioLegend	200
IFN-γ	BV421	505838	BioLegend	200
TNF-α	PE	506306	BioLegend	200
IL-2	APC	503810	BioLegend	200
TOX	PE	12-6502-82	eBioscience	100
GFP	GFP	A21311	Invitrogen	200
CellTrace Violet	BV421	C34557	Invitrogen	10 µM

Western blot antibodies			
Target	Catalog number	Provider	Dilution
CASP8	4927S	Cell Signaling Technology	1,000
Cleaved CASP8	8592S	Cell Signaling Technology	1,000
CASP3	9662S	Cell Signaling Technology	1,000
Cleaved CASP3	9661S	Cell Signaling Technology	1,000
Vinculin	14-9777-82	Thermo Fisher Scientific	1,000
NDRG3	5846S	Cell Signaling Technology	1,000
LDHA	2012S	Cell Signaling Technology	1,000
c-FLIP	56343T	Cell Signaling Technology	1,000
BIM	2933T	Cell Signaling Technology	1,000
Nur77	554088	BD Biosciences	250
Anti-rabbit HRP (Horseradish Peroxidase)	31460	Thermo Fisher Scientific	5,000
Anti-mouse HRP (Horseradish Peroxidase)	58802S	Cell Signaling Technology	1,000

sgRNA sequences		
Name	Target	Sequence
Scr #1	Nontargeting	GCACUACCAGAGCUAACUCA
*Ndrg3*_001	NDRG3	CAUGACAUUGAAACACCGCA
*Ndrg3*_002	NDRG3	CUUAAGUGUGUGAGAACAGG
*Ldha*_001	LDHA	CAAGCUGGUCAUUAUCACCG
*Ldha*_002	LDHA	GUUGCAAUCUGGAUUCAGCG
*Fas*_001	FAS	GAGGACUGCAAAAUGAAUGG
*Fas*_002	FAS	CAGUUAAGAGUUCAUACUCA
*Fas*_003	FAS	UAUUUAUAUAUCGAAAGUAC
*Fas*_004	FAS	CAUUUGCAUACUCACACGAC
		

shRNA sequences		
Name	Target	Full hairpin sequence
*Ndrg3*_shRNA	*Ndrg3*	tgctgttgacagtgagcgaagagatcaaaccgcttctaaatagtgaagccacagatgtatttagaagcggtttgatctctgtgcctactgcctcgga
NT_shRNA	*Nontargeting*	tgctgttgacagtgagcgaaggcagaagtatgcaaagcattagtgaagccacagatgtaatgctttgcatacttctgcctgtgcctactgcctcgga

General research materials		
Product	Catalog number	Provider
eBioscience Foxp3/Transcription Factor Staining Buffer Set	00-5523-00	Thermo Fisher Scientific
Fixation Buffer	420801	BioLegend
Lonza P3 kit	V4XP-3032	Lonza
S.p. Cas9 Nuclease V3	1081059	Integrated DNA Technologies
Goat anti-Mouse IgG (HandL) – Affinity Pure, HRP conjugate	72-8042-M001	Jackson ImmunoResearch
Anti-CD3e (Purified NA/LE)	553057	BD Biosciences
Anti-CD28 (Purified)	553295	BD Biosciences
IL-2 (recombinant, murine)	212-12	PeproTech
IL-7 (recombinant, murine)	217-17	PeproTech
IL-15 (recombinant, murine)	210-15	PeproTech
RPMI 1640 Medium	11875093	Thermo Fisher Scientific
Dulbecco’s Modified Eagle Medium	11965092	Gibco/Thermo Fisher Scientific
L-glutamine	25030081	Thermo Fisher Scientific
Penicillin-streptomycin	15140122	Gibco/Thermo Fisher Scientific
Geneticin	10131027	Invitrogen
Trypsin-EDTA (0.25%), phenol red	25-200-056	Thermo Fisher Scientific
PMA	P1585	MilliporeSigma
Ionomycin	9995S	Cell Signaling Technology
DMSO (dimethyl sulfoxide)	25-950-CQC	Corning
ACK buffer	RGF3015	Thermo Fisher Scientific
Collagenase type IV	C5138	Millipore Sigma
DNAse1	10104159001	Millipore Sigma/Roche
Mojosort Streptavidin Nanobeads	480016	BioLegend
Anti-TER119—Biotin	116204	BioLegend
Anti-CD4—Biotin	100508	BioLegend
Anti-CD11b—Biotin	101204	BioLegend
Anti-CD11c—Biotin	117304	BioLegend
Anti-B220—Biotin	103204	BioLegend
Anti-CD49b—Biotin	108904	BioLegend
Anti-MHCII—Biotin	107604	BioLegend
Anti-TCRγδ	553176	BD Biosciences
FASLG blocking antibody	106612	BioLegend
X-tremeGENE 9	6365779001	MilliporeSigma
Polybrene	TR-1003	MilliporeSigma
RIPA Lysis and Extraction buffer	89900	Thermo Fisher Scientific
Pierce Protease Inhibitor Tablets	A32963	Thermo Fisher Scientific
4%–15% Mini-PROTEAN TGX Precast Protein Gels	4561086	Bio-Rad
SuperSignal West Pico PLUS Chemiluminescent Substrate	34580	Thermo Fisher Scientific
Laemmli sample buffer, 4×	1610747	Bio-Rad

### In vitro cytokine stimulations and intracellular cytokine staining

Tumor cell suspensions were stimulated in T cell media (RPMI with 10% FBS, penicillin-streptomycin, L-glutamine, 50 µM β-mercaptoethanol) in the presence of ionomycin (Cell Signaling Technology; final concentration of 1 μg/mL), PMA (Sigma-Aldrich; final concentration of 50 ng/mL), and brefeldin A for 5 h at 37 °C. Cells were stained for viability and surface markers as described previously, and then fixed with fixation buffer (BD) for 10 min at room temperature. Cells were permeabilized with eBioscience Foxp3/Transcription Factor Staining kit fixation/permeabilization buffer for 45 min on ice. Intracellular cytokine staining was performed in 1× permeabilization buffer (BD) for 45 min on ice.

### Adoptive T cell transfer

Spleens and lymph nodes were isolated from naïve P14^+^ mice. Spleens and lymph nodes were dissociated with 1 mL syringe plungers over a 70 µm nylon strainer. Naïve P14^+^ T cells were isolated through negative selection by incubating cell suspensions with biotinylated antibodies against CD4, B220, MHCII, CD11b, CD11c, CD49b, TCRγδ, and TER119 in MACS buffer. MojoSort beads were added at 10% v/v for 5 min before placing the cell suspension on a magnet and collecting the supernatant. For CRISPR-Cas9 ribonucleoprotein (RNP) electroporation experiments, naïve P14^+^ T cells were electroporated, rested for 20 min, then P14^+^ T cells were injected into recipient mice 18 to 24 h before infection. For T cell cotransfer experiments, 5 × 10^4^ P14^+^ T cells of each genotype (*sgScr* and experimental) were transferred when infecting with LCMV-Armstrong and 5 × 10^3^ P14^+^ T cells of each genotype were transferred when infecting with LCMV-Clone 13. For homeostatic proliferation experiments, 1 × 10^6^ P14^+^ T cells of each genotype were transferred into *Rag1*^−/−^ mice. For single genotype transfer experiments, 1 × 10^5^ SMARTA^+^ T cells were transferred when infecting with LCMV-Armstrong. For viral transduction experiments, naïve P14^+^ T cells were activated in vitro for 18–22 h before transduction. P14^+^ T cells that were successfully transduced were sorted 18 h after transduction and 5 × 10^5^ transduced P14^+^ T cells were injected into recipient mice directly after sorting.

### Retrovirus production and transduction

Retrovirus was prepared by transfection of HEK293T cells with pCL-Eco and *Ndrg3* overexpression (OE), empty vector (EV), *Ndrg3* knockdown (*Ndrg3* short hairpin RNA [shRNA]), or nontargeting shRNA (NT shRNA) plasmids using XtremeGene9 transfection reagent. For transductions, P14^+^ T cells were stimulated for 18 to 22 h with plate-bound 2 µg/mL anti-CD3, 1 µg/mL anti-CD28, and 10 ng/mL murine recombinant IL-2. Retroviral supernatants supplemented with polybrene (10 µg/mL) were added to activated T cell suspensions, and then cells were centrifuged for 90 min at 1500 *g* at 30 °C in 12-well plates. The brake speed of the centrifuge was reduced to avoid disturbing the retroviral infection process. Cells were incubated for 3 h at 37 °C after centrifugation. They were then washed and returned to 12-well plates in T cell media with 10 ng/mL recombinant murine IL-2.

### In vitro restimulation protocol

At day 0, MACS-purified naïve T cells underwent CRISPR-Cas9 RNP electroporation and were then activated with plate-bound 2 µg/mL anti-CD3, 1 µg/mL anti-CD28, and 10 ng/mL murine recombinant IL-2 in T cell media. At day 1, T cells remained on the plate-bound stimulation. At day 2, T cells were removed from the plate-bound stimulation in the morning. They were then washed and resuspended in T cell media with 10 ng/mL murine recombinant IL-2. At day 3, T cells were removed from culture wells and counted. A total of 6 × 10^5^ T cells per experimental condition were restimulated for 6 h with plate-bound 2 µg/mL anti-CD3 and 10 ng/mL murine recombinant IL-2 in T cell media. After 6 h of restimulation, T cells were washed and resuspended in T cell media with 10 ng/mL murine recombinant IL-2. At day 4, T cells remained in culture with T cell media and 10 ng/mL murine recombinant IL-2. At day 5, T cells were removed from culture wells and were restimulated for 6 h with plate-bound 2 µg/mL anti-CD3 and 10 ng/mL murine recombinant IL-2 in T cell media. After 6 h of restimulation, T cells were washed and resuspended in T cell media with 10 ng/mL murine recombinant IL-2. At day 6, T cells were analyzed by flow cytometry and protein was harvested for Western blot.

Experiments that included the use of a FASLG blocking antibody initiated treatment with the blocking antibody at the time of stimulation on day 3 at 7.5 µg/mL and continued at that concentration until the end of the assay.

Assay quantification parameters were that cell counts were taken on day 3 before the first restimulation, on day 5 before the secondary restimulation, and on day 6 at the end of the assay. Flow cytometry samples were taken before and after restimulation on days 3 and 5 as well as on day 6 in coculture experiments. T cells that did not receive restimulation during the assay were kept in accompanying wells with culture media changes happening at the same time as in the restimulated condition. All quantification steps were also carried out for the not restimulated group.

### CRISPR-Cas9 RNP electroporation

Cas9/single guide RNA (sgRNA) mixtures were prepared (0.6 µL 62 nM recombinant Cas9, 0.3 nmol sgRNA, 3.4 µL RNase-free H_2_O) and incubated for 10 min at room temperature. A total of 2 to 3 × 10^6^ MACS-purified naïve P14^+^ cells were washed in PBS and suspended in 20 µL supplemented P3 buffer (Lonza; 16.4 µL P3 buffer and 3.6 µL supplement 1). Resuspended cells were immediately mixed with 5 µL Cas9/sgRNA mixture and transferred to a Lonza nucleofector strip. Cells were electroporated using the program DN100. After electroporation, 5 mL of prewarmed T cell media was added to the cells and they were then rested for 20 min at 37 °C. Cells were washed extensively with T cell media prior to transfer into recipient mice.

### Western blotting

Cells were lysed in RIPA buffer with Pierce protease inhibitor (A32963). Cells were lysed at a density of 5 × 10^5^ cells/10 µl for 10 min on ice. Lysates were centrifuged for 10 min at 12000 g and the supernatant was collected. The appropriate volume of 4× Laemmli sample buffer and β-mercaptoethanol (final concentration 2.5% v/v) was added to lysates and boiled at 95 °C for 5 min. Boiled lysates were stored at −80°C. Western blotting was performed using a NuPAGE electrophoresis system. Samples were loaded onto a 4% to 15% gradient acrylamide gel and electrophoresis was performed at 100 V for 60 min. Protein from the gel was transferred to a PVDF membrane at 100 V for 90 min. PVDF membranes were activated in methanol before initiating the transfer. After transferring the protein to PVDF membranes, the membranes were blocked in a solution of 5% w/v bovine serum albumin in phosphate-buffered saline with 0.05% tween 20 (PBST) at room temperature for 60 min. The blocked membranes were stained with primary antibodies (Table 1) overnight. The following day, the membranes were washed 3 times for 10 min with PBST before being incubated with secondary antibodies conjugated to horseradish peroxidase for 60 min. Membranes were washed with PBST again 3 times before they were developed using SuperSignal West Pico PLUS Chemiluminescent Substrate (Thermo Fisher Scientific 34580).

### Cell proliferation quantification

For assessment of proliferation, T cells were labeled with CellTrace Violet (CTV) (Thermo Fisher Scientific) according to the manufacturer’s instructions and cultured with 10 ng/mL murine recombinant IL-2 (PeproTech). Naïve T cells were labeled with CTV before plate-bound activation. Cells were cultured for 4 d after initial activation and then collected for analysis.

### Statistics

All statistical analyses were performed using GraphPad Prism (version 10; GraphPad Software). Specific statistical tests are identified in the figure legends for clarity. *P* values that were <0.05 were considered significant and *P* values were displayed on figures as such: *P < 0.05, **P < 0.01, ***P < 0.001, and ****P < 0.0001. In general, a Student’s *t* test was used to compare 2 populations when only 1 time point was being analyzed. The homeostatic proliferation model, the tumor challenge model, and comparing tumor mass are examples of experiments in which a Student’s *t* test was used. For experiments in which multiple time points were collected for a 2-population comparison, a 2-way analysis of variance (ANOVA) test with Šidák’s multiple comparisons correction was performed to assess the *P* value for each population comparison at each time point. The experiments that this analysis was performed on were the time courses for the chronic infection model, the acute infection model, the in vitro restimulation model, and the tumor control model. A 1-way ANOVA with Tukey’s multiple comparisons correction was used to compare 3, or more, populations in a bar graph. Comparisons of the proportion of live and dead cells at the end of the in vitro restimulation model are examples of assessments in which the 1-way ANOVA analysis was applied.

## Results

### Section 1: NDRG3 is essential to sustain antiviral and antitumor T cell responses

**Figure 1:**
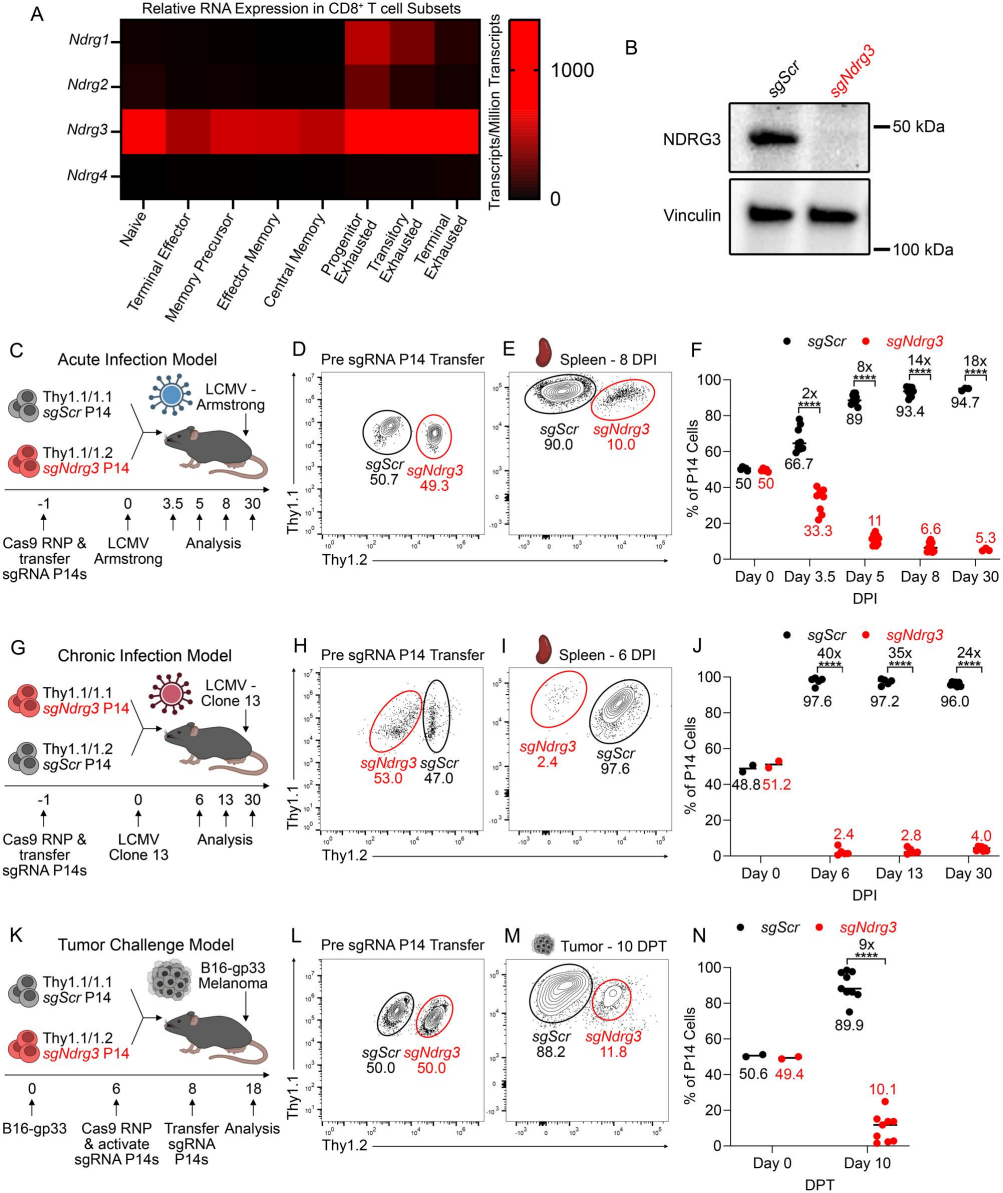
NDRG3 is essential to sustain antiviral and antitumor T cell responses. (A) Expression of *Ndrg1*, *Ndrg2*, *Ndrg3*, and *Ndrg4* messenger RNA in T cell subsets in different infection contexts. Data from the Immunological Genome Project and Hudson et al. (National Center for Biotechnology Information Sequence Read Archive PRJNA497086).^44^ (B) Western blot showing NDRG3 levels in *sgScr* and *sgNdrg3* P14^+^ cells that had been cultured in vitro for 4 d after initial Cas9 RNP + sgRNA electroporation and activation. Western blot is representative of 5 independent biological replicates. (C) Experimental schematic of cotransfer experiment in which *sgScr* and *sgNdrg3* P14^+^ cells with different Thy1 congenic markers were mixed at a 1:1 ratio then transferred into B6 recipient mice that were subsequently infected with LCMV-Armstrong 1 d later. (D, E) Representative flow plots showing the frequency of *sgScr* and *sgNdrg3* P14^+^ cells pretransfer, to ensure a 1:1 ratio (D), and in the spleen 8 DPI with LCMV-Armstrong (E). Each plot is gated on Thy1.1^+^ P14^+^ T cells showing Thy1.1 and Thy1.2 expression to identify the different cell populations as indicated. (F) Quantification of the Continuedfrequency of splenic *sgScr* and *sgNdrg3* P14^+^ cells pretransfer and at 3.5, 5, 8, and 30 DPI with LCMV-Armstrong. Data are pooled across 5 independent biological replicates (n = 9 for day 3.5, n = 10 for day 5, n = 9 for day 8, and n = 3 for day 30). (G) Experimental schematic of cotransfer experiment in which *sgScr* and *sgNdrg3* P14^+^ cells with different Thy1 congenic markers were mixed at a 1:1 ratio then transferred into B6 recipient mice that were subsequently infected with LCMV-Clone 13 1 d later. (H, I) Representative flow plots showing the frequency of *sgScr* and *sgNdrg3* P14^+^ cells pretransfer (H) and in the spleen 6 DPI with LCMV-Clone 13 (I). (J) Quantification of the frequency of *sgScr* and *sgNdrg3* P14^+^ cells pretransfer and in the spleen at 6, 13, and 30 DPI with LCMV-Clone 13. Data are pooled across 2 independent biological replicates (n = 5 for day 6, n = 5 for day 13, and n = 7 for day 30). (K) Experimental schematic of cotransfer experiment in which *sgScr* and *sgNdrg3* P14^+^ cells with different Thy1 congenic markers were mixed at a 1:1 ratio then transferred into B6 recipient mice that were implanted with subcutaneous B16-gp33 melanomas 8 d prior. (L, M) Representative flow plots showing the frequency of *sgScr* and *sgNdrg3* P14^+^ cells pretransfer (L) and TILs 10 d post-transfer (DPT) into tumor-bearing mice (M). (N) Quantification of the frequency of *sgScr* and *sgNdrg3* P14^+^ cells pretransfer and TILs 10 DPT. Data are pooled across 2 independent biological replicates (n = 9). Fold changes that are represented in panels F, J, and N are calculated using (the frequency of *sgScr* P14+ cells)/(the frequency of *sgNdrg3* P14^+^ cells). Two-way ANOVA tests with Šidák’s multiple comparisons correction were performed to assess comparisons between *sgScr* and *sgNdrg3* at each time point in the LCMV-Armstrong and LCMV-Clone 13 settings. A 2-sided Student’s *t* test was used in the B16-gp33 setting. *P < 0.05, **P < 0.01, ***P < 0.001, ****P < 0.0001, ns, not significant.

Upon assessing the expression levels of the different NDRG family members in T cells under various immunological challenge contexts, we noticed that *Ndrg3* was the only NDRG family member that was highly expressed in CD8^+^ T cells across multiple challenge models (acute infection and chronic infection) in all T cell differentiation subsets (Fig. 1A).^43^^,^^44^ Given that NDRG3 does not have a reported role in regulating antigen-specific T cell responses, we decided to further investigate the role of NDRG3 in regulating T cell biology. To achieve this, we utilized a genetic knockout (KO) approach and deleted NDRG3 in naïve T cells by delivering guide RNA and recombinant Cas9 protein through Cas9 RNP (CRISPR-Cas9 RNP) electroporation.^45^ This method consistently provided over a 95% reduction in NDRG3 protein level when assessing the bulk electroporated T cell population (Fig. 1B). To assess how the loss of NDRG3 affects the behavior of antigen-specific T cell responses, NDRG3 was deleted in naïve P14^+^ CD8^+^ T cells, which have a transgenic TCR that recognizes the gp_33-41_ epitope (referred to as gp33) of LCMV. NDRG3 KO P14^+^ T cells (referred to as *sgNdrg3* P14^+^ cells) were then adoptively transferred into naïve C57BL/6 (B6) mice at a 1:1 ratio with congenically distinct control P14^+^ T cells that received a nontargeting scramble sgRNA (referred to as *sgScr* P14^+^ cells) (Fig. 1C, D). These mice were subsequently infected with LCMV-Armstrong, a model of acute viral infection, 24 h after adoptive transfer of the sgRNA P14^+^ cells. The spleens of the mice were then harvested for analysis at 3.5, 5, 8, and 30 d post-infection (DPI).

The *sgNdrg3* P14^+^ cells displayed profound defects in clonal expansion compared with the *sgScr* P14^+^ cells over the first week of infection. At 3.5 DPI, the *sgNdrg3* P14^+^ cells comprised 33% of the transferred sgRNA P14^+^ cell population, reflecting a 2-fold reduction in frequency of *sgNdrg3* P14^+^ cells (Fig. 1F). This 2-fold reduction is seen in the change in the ratio of *sgNdrg3* frequency to *sgScr* frequency as it shifted from 50%:50% (1:1) at 0 DPI to 33%:67% (1:2) at 3.5 DPI (Fig. 1D, F). The capacity for clonal expansion of the *sgNdrg3* P14^+^ cells rapidly declined after 3.5 DPI as just 2 d later, at 5 DPI, the *sgNdrg3* P14^+^ cells made up only 11% of the donor sgRNA P14^+^ cell population, representing an 8-fold reduction in frequency of the *sgNdrg3* P14^+^ cells (Fig. 1F). The *sgNdrg3* P14^+^ cells continued to show drastically impaired expansion till the peak of the CD8^+^ T cell response at 8 DPI as they were reduced by 14-fold and made up only 6% to 7% of the sgRNA P14^+^ cell population (Fig. 1E, F). Beyond this point, however, there was minimal further attrition of the *sgNdrg3* P14^+^ cells as they represented 5% of the sgRNA P14^+^ cell pool at 30 DPI (Fig. 1F). To confirm these findings using an alternative method of disrupting NDRG3 function in T cells, we assessed whether an shRNA-mediated knockdown of *Ndrg3* would yield a similar effect to the genetic KO approach of *Ndrg3* (Fig. S1A, B). By 7 d post LCMV-Armstrong infection, the *Ndrg3* shRNA P14^+^ cells made up only 10% of the transferred shRNA P14^+^ cell population in the spleen, representing a 9-fold reduction (Fig. S1C–E) and aligning closely with the strong effects seen in the genetic KO approach. Together, these results demonstrate that NDRG3 is critical for antigen-specific CD8^+^ T cells to expand normally during a response to an acute viral infection.

Given the strong impact of NDRG3-deficiency on CD8^+^ T cell expansion, we next asked whether CD4^+^ T cells were similarly affected. NDRG3 was deleted using the CRISPR-Cas9 RNP method in SMARTA^+^ CD4^+^ T cells, which recognize a different epitope of LCMV-Armstrong (gp_61-80_). Then 100,000 *sgNdrg3* SMARTA^+^ cells were transferred into naïve B6 recipient mice and an equivalent number of *sgScr* SMARTA^+^ cells were transferred into parallel control mice (Fig. S1F). These recipient mice were then infected with LCMV-Armstrong 24 h later. At 8 DPI, the *sgNdrg3* SMARTA^+^ cells made up only 6 percent of the total CD4^+^ T cell population whereas the *sgScr* SMARTA^+^ cells made up nearly 65 percent of the total CD4^+^ T cell population (Fig. S1G, H). This 11-fold reduction in *sgNdrg3* SMARTA^+^ cells demonstrates that NDRG3 deficiency also profoundly impairs CD4^+^ T cell clonal expansion during an acute viral infection.

Because NDRG3 was highly expressed in T cells in other pathogenic challenge contexts, we also cotransferred *sgNdrg3* and *sgScr* P14^+^ cells into a model of chronic viral infection (LCMV-Clone 13) and a tumor challenge model (B16-gp33) to assess NDRG3’s role in regulating T cell biology in these other important immunological challenge settings (Fig. 1G, K). Strikingly, in the LCMV-Clone 13 setting, the *sgNdrg3* P14^+^ cells were already reduced to 2–3% of the transferred sgRNA P14^+^ cell pool at 6 DPI, reflecting a 40-fold reduction relative to *sgScr* P14^+^ cells (Fig. 1H–J). Similarly, the *sgNdrg3* P14^+^ cells in the B16-gp33 tumor challenge constituted only 10% of the transferred sgRNA P14^+^ cell population in the tumor 10 d post-transfer, showing a 9-fold reduction compared with the *sgScr* P14^+^ cells (Fig. 1L–N). Altogether, these data demonstrate that NDRG3 is indispensable for sustaining antigen-specific T cell responses across a variety of immunological challenge contexts.

### Section 2: NDRG3 marginally impacts T cell survival in contexts with limited restimulation

**Figure 2:**
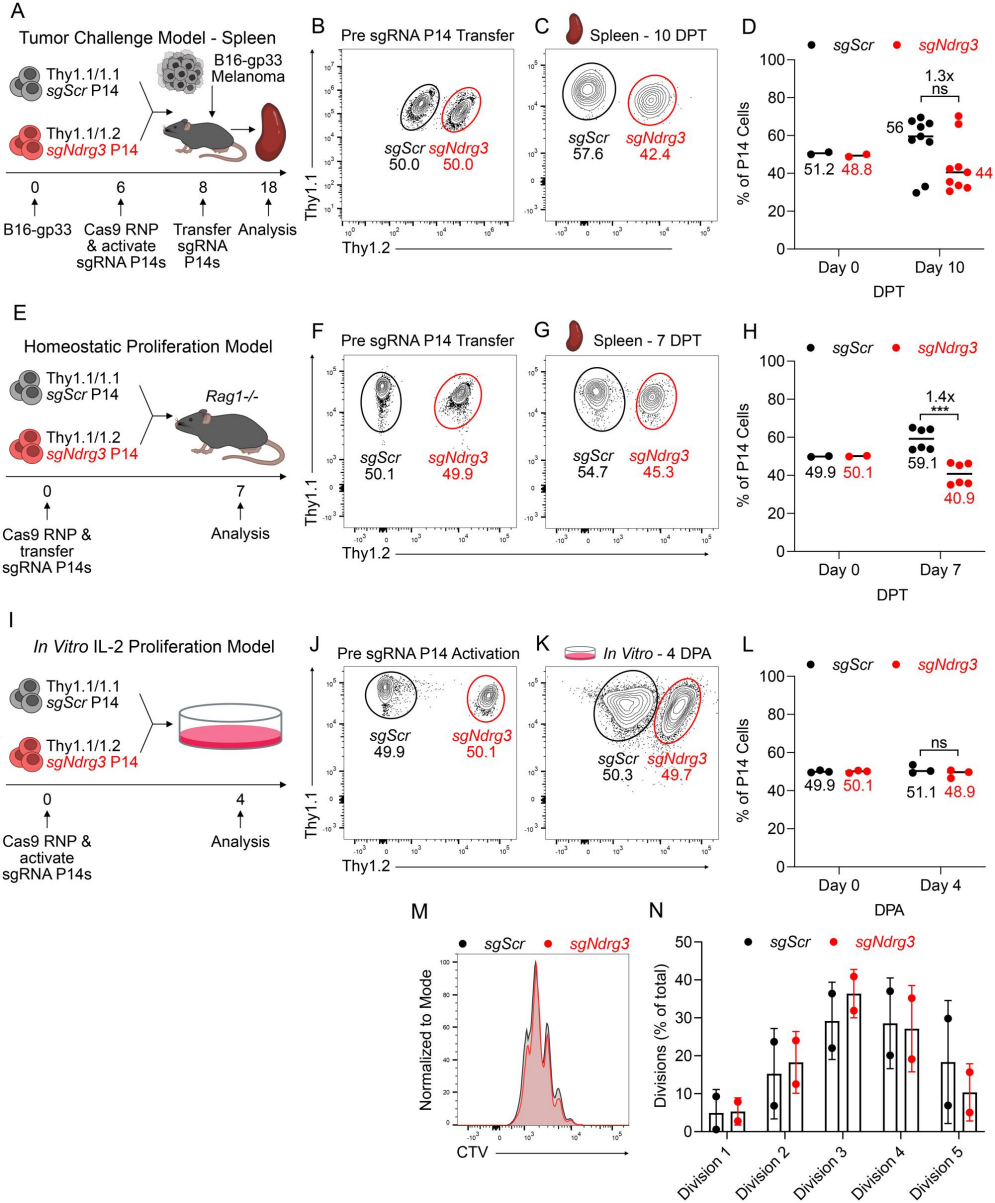
NDRG3 marginally impacts T cell survival in contexts with limited restimulation. (A) Experimental schematic of cotransfer experiment where *sgScr* and *sgNdrg3* P14^+^ cells with different Thy1 congenic markers were mixed at a 1:1 ratio then transferred into B6 recipient mice that were implanted with subcutaneous B16-gp33 melanoma 8 d prior. (B, C), Representative flow plots showing the frequency of *sgScr* and *sgNdrg3* P14^+^ cells pretransfer (B) and in the spleen 10 d post-transfer (DPT) into tumor-bearing mice (C). Each plot is gated on Thy1.1^+^ P14^+^ T cells showing Thy1.1 and Thy1.2 expression to identify the different cell populations as indicated. (D) Quantification of the frequency of *sgScr* and *sgNdrg3* P14^+^ cells pretransfer and in the spleen 10 DPT into tumor-bearing mice. Data are pooled across 2 independent biological replicates (n = 9). (E) Experimental schematic of cotransfer experiment where *sgScr* and *sgNdrg3* P14^+^ cells with different Thy1 congenic markers were mixed at a 1:1 ratio then Continuedtransferred into *Rag1*^−/−^ recipient mice. (F, G) Representative flow plots showing the frequency of *sgScr* and *sgNdrg3* P14^+^ cells pretransfer (F) and in the spleen 7 DPT (G). (H) Quantification of the frequency of *sgScr* and *sgNdrg3* P14^+^ cells pretransfer and in the spleen 7 DPT into *Rag1*^−/−^ mice. Data are pooled across 2 independent biological replicates (n = 6). (I) Experimental schematic of coculture experiment in which *sgScr* and *sgNdrg3* P14^+^ cells with different Thy1 congenic markers were mixed at a 1:1 ratio, activated with a plate-bound anti-CD3 and anti-CD28 stimulation, and then cultured in 10 ng/mL IL-2 in vitro for 4 d. (J, K) Representative flow plots showing the frequency of *sgScr* and *sgNdrg3* P14^+^ cells preactivation (J) and 4 d post-activation (DPA) in vitro (K). (L) Quantification of the frequency of *sgScr* and *sgNdrg3* P14^+^ cells preactivation and 4 DPA in vitro. Data are pooled across 3 independent biological replicates. (M) CTV staining of *sgScr* and *sgNdrg3* P14^+^ cells cultured for 4 d in vitro in 10 ng/mL IL-2. (N) Quantification of proportion of dividing cells in each of the *sgScr* and *sgNdrg3* P14^+^ cell groups. Data are pooled across 2 independent biological replicates, and a 2-sided Student’s *t* test was used to assess these experimental settings. Fold changes that are represented in panels D and H are calculated using (the frequency of *sgScr* P14+ cells)/(the frequency of *sgNdrg3* P14^+^ cells). *P < 0.05, **P < 0.01, ***P < 0.001, ****P < 0.0001, ns, not significant.

In the tumor challenge model (Fig. 1K–N), we also analyzed paired spleen samples from each animal. Unlike within the tumor, in which *sgNdrg3* P14^+^ cells were reduced by 9-fold, the *sgNdrg3* and *sgScr* P14^+^ cells were present at nearly equal frequencies in the spleen (44% vs. 56%) (Fig. 2A–D). Given the large divergence in the relative prevalence of *sgNdrg3* P14^+^ cells between the spleen and tumor contexts in this model, we hypothesized that the *sgNdrg3* P14^+^ cells persisted better in the spleen due to low levels of exposure to tumor antigens. To test this in a more controlled manner, we assessed whether *sgNdrg3* P14^+^ cells could survive similarly well to control T cells in a setting of T cell proliferation that does not rely on antigen stimulus. Thus, we utilized a lymphopenia-induced proliferation model (often referred to as homeostatic proliferation) in which naïve T cells are transferred into lymphopenic hosts to induce proliferation in an antigen-independent manner.^46–51^ To achieve this, *sgNdrg3* P14^+^ cells were cotransferred at a 1:1 ratio with *sgScr* P14^+^ cells into *Rag1*^−/−^ mice (Fig. 2E, F). After 1 wk of lymphopenia-induced proliferation, the *sgNdrg3* P14^+^ cells comprised 41% of the donor sgRNA P14^+^ cell population (Fig. 2G, H), reflecting only a modest reduction in their relative frequency compared with the *sgScr* P14^+^ cells in this setting of antigen-free T cell proliferation.

We further assessed this phenomenon by culturing activated *sgNdrg3* P14^+^ cells in IL-2 for 4 d in vitro to assess IL-2 driven T cell proliferation. In this model, *sgNdrg3* and *sgScr* P14^+^ cells were initially activated with a plate-bound anti-CD3 and anti-CD28 stimulation for 24 h, then cultured in 10 ng/mL IL-2 for 72 h without any TCR restimulation (Fig. 2I, J). In this setting, the *sgNdrg3* P14^+^ cells persisted at an almost equal frequency compared with the *sgScr* P14^+^ cells (49%:51%, *sgNdrg3: sgScr*) (Fig. 2K, L). Naïve sgRNA P14^+^ cells were also labeled with CTV to measure proliferative rate through CTV dilution. The *sgNdrg3* P14^+^ cells diluted out the CTV at an almost identical rate as the *sgScr* P14^+^ cells (Fig. 2M, N), showing that the loss of NDRG3 had minimal effects on proliferative rate in this setting. Overall, these data support the conclusion that NDRG3-deficiency has only a modest effect on T cell proliferation and survival in contexts with limited to no repeated antigen stimulus.

### Section 3: TCR restimulation eliminates T cells lacking NDRG3

**Figure 3:**
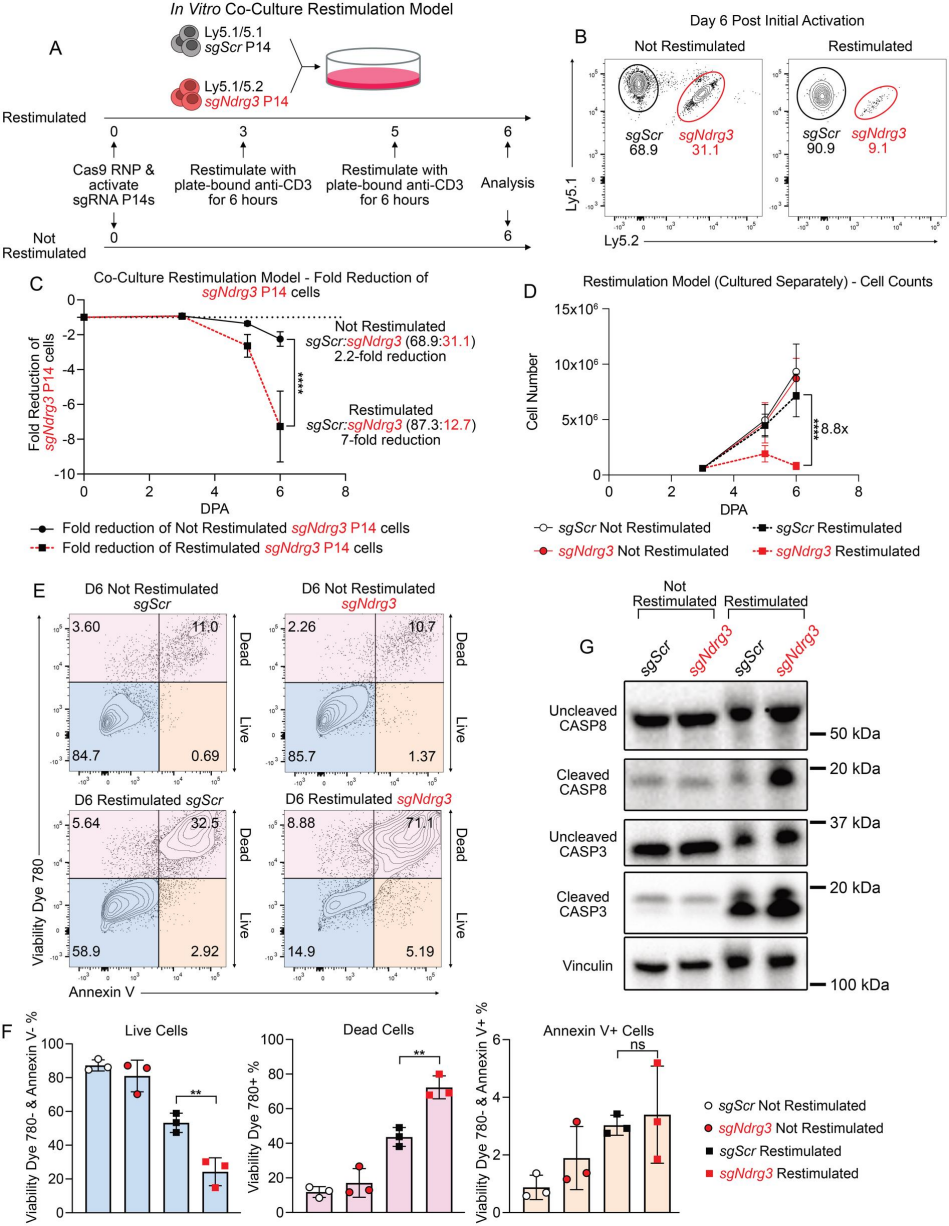
NDRG3 protects T cells from restimulation-induced cell death. (A) Experimental schematic of coculture in vitro restimulation assay in which *sgScr* and *sgNdrg3* P14^+^ cells with different Ly5 congenic markers were mixed at a 1:1 ratio, activated with a plate-bound anti-CD3 and anti-CD28 stimulation, and then cultured in vitro for 6 d in 10ng/mL IL-2, in which one group was restimulated twice with anti-CD3 and the other group was not restimulated. (B) Representative flow plots showing the frequency of *sgScr* and *sgNdrg3* P14^+^ cells at 6 d post-activation (DPA) in the not restimulated group (left) and the restimulated group (right). Each plot is gated on Ly5.1^+^ P14^+^ T cells showing Ly5.1 and Ly5.2 expression to identify the different cell populations as indicated. (C) Quantification of the fold reduction of the *sgNdrg3* P14^+^ cells relative to the *sgScr* P14^+^ cells for both experimental groups (not restimulated [black line] and restimulated [red dashed line]). Fold reduction is calculated as –1 × (*sgScr* frequency/*sgNdrg3* frequency). ContinuedData are pooled across 3 independent biological replicates. (D) Quantification of the number of cells present in the in vitro restimulation assay in which the experimental groups were cultured separately at 3, 5, and 6 DPA. Data are pooled across 3 independent biological replicates. (E, F) Cells from the restimulation assay were stained at 6 DPA with Viability Dye 780 and Annexin V to identify live cells (Viability Dye 780^−^ and Annexin V^−^), dead cells (Viability Dye 780^+^), and Annexin V^+^ cells (Viability Dye 780^−^ and Annexin V^+^) as shown in representative flow plots (E) and quantified in bar graphs (F). Data are pooled across 3 independent biological replicates. (G) Western blot showing uncleaved CASP8, cleaved CASP8, uncleaved CASP3, cleaved CASP3, and vinculin in *sgScr* and *sgNdrg3* P14^+^ cells at 6 DPA in the in vitro restimulation assay. Western blot is representative of 3 independent experiments. A 2-way ANOVA with Šidák’s multiple comparisons correction was performed to assess each comparison between *sgScr* and *sgNdrg3* in both the coculture and single-genotype culture restimulation assays. A 1-way ANOVA with Tukey’s multiple comparisons correction was used to analyze the proportions of live, dead, and Annexin V^+^ cells across the different experimental conditions. *P < 0.05, **P < 0.01, ***P < 0.001, ****P < 0.0001, ns, not significant.

The loss of NDRG3 compromises T cell expansion in contexts with repeated antigen stimulation (acute viral infection, chronic viral infection, tumor challenges) but has a minimal effect on T cell survival and proliferation in contexts with limited antigen restimulation. These observations suggested that T cell restimulation could be a contributing factor to the reduction in frequency of the *sgNdrg3* P14^+^ cells in the repeated antigen exposure settings; thus, we developed an in vitro system of repeated TCR restimulations to more directly test this hypothesis (Fig. 3A). After the initial plate-bound anti-CD3 and anti-CD28 activation, cocultured *sgNdrg3* and *sgScr* P14^+^ cells were restimulated with plate-bound anti-CD3 for 6 h at 3 and 5 d after initial activation. A paired set of *sgNdrg3* and *sgScr* P14^+^ cells were also cultured in parallel that did not receive any TCR restimulation. The final analysis was performed at day 6 post–initial activation. Strikingly, the restimulated *sgNdrg3* P14^+^ cells comprised only 12% of the coculture at the end point of this assay, reflecting a 7-fold reduction in frequency compared with the restimulated *sgScr* P14^+^ cells (Fig. 3B, C; Fig. S2A). In contrast, the experimental group that was not restimulated saw a relatively modest 2-fold reduction in the prevalence of the *sgNdrg3* P14^+^ cells, as the *sgNdrg3* P14^+^ cells made up 31% of the coculture (Fig. 3B, C; Fig. S2A). The greater loss of the *sgNdrg3* P14^+^ cells in the restimulated condition compared with the not restimulated condition (7-fold vs 2-fold) strengthens the observation that NDRG3 is necessary for T cell survival under repetitive TCR stimulation and suggests that the process of being restimulated drives the decline of the NDRG3-deficient T cells. We also performed the restimulation assay with *sgNdrg3* and *sgScr* P14^+^ cells cultured separately and the results closely matched the coculture system, wherein the restimulated *sgNdrg3* P14^+^ cells were reduced in cell number by 9-fold compared with the restimulated *sgScr* P14^+^ cells (Fig. 3D). Interestingly, the restimulated *sgNdrg3* P14^+^ cells decreased in total cell number after the last restimulation, suggesting that the restimulated *sgNdrg3* P14^+^ cells were undergoing cell death after restimulation. Given that RICD is a well-established program present in T cells that induces cell death upon TCR restimulation, these findings suggest that NDRG3-deficient T cells could be perishing due to exaggerated RICD.

### Section 4: NDRG3 protects T cells from restimulation-induced cell death

To more closely assess whether *sgNdrg3* P14^+^ cells had enhanced RICD compared with *sgScr* P14^+^ cells, we examined the proportion of live vs. dead cells by flow cytometry at day 6 of the restimulation assay to determine if the *sgNdrg3* P14^+^ cells were undergoing more frequent cell death. We observed a substantial increase in the proportion of dead cells (Viability Dye 780^+^) in the restimulated *sgNdrg3* P14^+^ cultures, with 72.3% of cells dying compared with 43.6% in restimulated *sgScr* P14^+^ cells (Fig. 3E, F). There was also a coordinate reduction in the proportion of live cells (Viability Dye 780^−^ and Annexin V^−^) in the restimulated *sgNdrg3* P14^+^ cultures, with only 24.3% of cells being alive compared with 53.4% in restimulated *sgScr* P14^+^ cells (Fig. 3E, F). In contrast, the survival capacity of the *sgNdrg3* and *sgScr* P14^+^ cells in the not restimulated condition was relatively similar (87% live in *sgScr*, 81% live in *sgNdrg3*) (Fig. 3E, F).

Having established that the *sgNdrg3* P14^+^ cells were dying more frequently upon restimulation, we next assessed the mechanism by which this was occurring. RICD is typically driven by FAS-mediated activation of CASP8, which can then activate executioner caspases like CASP3.^17^^,^^52^^,^^53^ Thus, we measured protein levels of uncleaved and cleaved CASP8 and CASP3 to look for an indication of CASP8 and CASP3 activation. We observed a markedly higher amount of cleaved CASP8 and an increase in cleaved CASP3 levels in the restimulated *sgNdrg3* P14^+^ cells compared with restimulated *sgScr* P14^+^ cells (Fig. 3G), supporting the notion that NDRG3-deficient T cells are more sensitive to RICD.

We also tested whether *sgNdrg3* P14^+^ cells were more sensitive to CWID, another canonical T cell death–inducing program. We found that neither reducing IL-2 culture concentration 100-fold for 12 h (Fig. S2B–D) nor switching to IL-7 plus IL-15 culture conditions (Fig. S2E–G) selectively reduced the frequency of the *sgNdrg3* P14^+^ cells compared with *sgScr* P14^+^ cells. These results support the hypothesis that enhanced RICD, and not CWID, compromises the survival capacity of T cells that lack NDRG3.

### Section 5: NDRG3 negatively regulates FAS signaling to protect T cells from RICD

**Figure 4:**
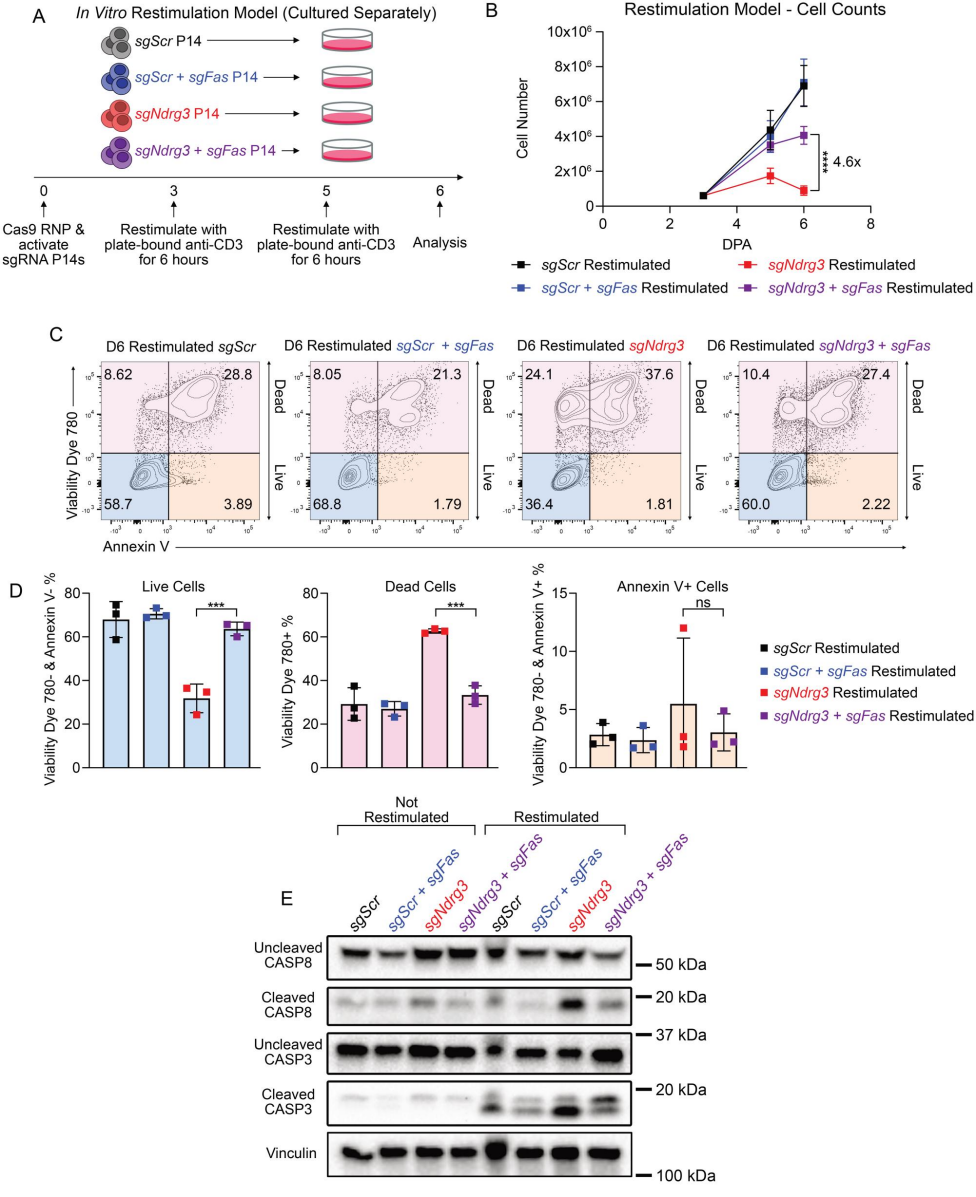
NDRG3 negatively regulates FAS signaling to protect T cells from RICD. (A) Experimental schematic of in vitro restimulation assay in which the experimental groups were cultured separately including *sgScr*, *sgScr + sgFas*, *sgNdrg3*, and *sgNdrg3 + sgFas* P14^+^ cells. (B) Quantification of the number of cells present in the in vitro restimulation assay at 3, 5, and 6 DPA. Data are pooled across 3 independent biological replicates. (C, D) Cells from the restimulation assay were stained at 6 DPA with Viability Dye 780 and Annexin V to identify live cells (Viability Dye 780^−^ and Annexin V^−^), dead cells (Viability Dye 780^+^), and Annexin V^+^ cells (Viability Dye 780^−^ and Annexin V^+^) as shown in representative flow plots (C) and quantified in bar graphs (D). Data are pooled across 3 independent biological replicates. (E) Western blot showing uncleaved CASP8, cleaved CASP8, uncleaved CASP3, Continuedcleaved CASP3, and vinculin in all genotypes at 6 DPA in the in vitro restimulation assay. Western blot is representative of 3 independent experiments. A 2-way ANOVA with Šidák’s multiple comparisons correction was performed to assess each comparison in the single-genotype culture restimulation assay. A 1-way ANOVA with Tukey’s multiple comparisons correction was used to analyze the proportions of live, dead, and Annexin V+ cells across the different experimental conditions. *P < 0.05, **P < 0.01, ***P < 0.001, ****P < 0.0001, ns, not significant.

Given the well-established link between FAS signaling and CASP8 activation, and the substantial increase in cleaved CASP8 observed in restimulated *sgNdrg3* P14^+^ cells, we next asked whether the heightened cell death seen in the restimulated *sgNdrg3* P14^+^ cells was dependent on FAS signaling. FAS was deleted in *sgNdrg3* and *sgScr* P14^+^ cells to generate *sgNdrg3 + sgFas* double KO (DKO) (purple) and *sgScr + sgFas* (FAS KO) (blue) cells (Fig. 4A; Fig. S2H, I). These cells were then cultured in the restimulation assay to compare the cell number and viability between the different experimental conditions (Fig. 4A). We observed that the loss of FAS in the *sgNdrg3 + sgFas* DKO P14^+^ cells increased the number of T cells by 4.6-fold compared with *sgNdrg3* P14^+^ cells at the final time point of the restimulation assay (Fig. 4B). Additionally, the deletion of FAS in the *sgNdrg3 *+* sgFas* DKO P14^+^ cells also rescued the proportion of live cells (Viability Dye 780^−^ and Annexin V^−^) from 32% in the *sgNdrg3* P14^+^ condition to 65% in the *sgNdrg3 *+* sgFas* DKO P14^+^ condition, which was notably similar to the survival frequency of the restimulated *sgScr* P14^+^ cells (Fig. 4C, D). Furthermore, the increase in cleaved CASP8 observed in the restimulated *sgNdrg3* P14^+^ cells was largely eliminated in the restimulated *sgNdrg3 + sgFas* DKO P14^+^ cells (Fig. 4E). We observed similar trends with an orthogonal approach using a FASLG blocking antibody, where the prevention of FAS ligation rescued both the cell number and survival of restimulated *sgNdrg3* P14^+^ cells (Fig. S3A–D). These results suggest that the loss of NDRG3 in T cells increases the susceptibility to FAS-mediated killing triggered by repetitive TCR restimulations and that NDRG3 operates as a negative regulator of FAS signaling in T cells.

To assess whether this increase in FAS-mediated RICD is due to altered expression of FAS pathway members in *sgNdrg3* P14^+^ cells, we assessed the abundance of uncleaved CASP8, FAS, FASLG, and c-FLIP at the conclusion of the in vitro restimulation assay. There was no significant difference in the abundance of any of these pathway members (Fig. 3G; Fig. S3E, F). Furthermore, we evaluated the expression of other regulators of cell viability (BIM, BCL2, and Nur77), which also showed minimal differences between restimulated *sgScr* and *sgNdrg3* P14^+^ cells (Fig. S3E, s3F). These findings indicate that NDRG3 likely does not alter RICD sensitivity through regulating the abundance of canonical cell viability modulators.

### Section 6: NDRG3 overexpression is sufficient to increase the tumor-controlling capacity of T cells

**Figure 5:**
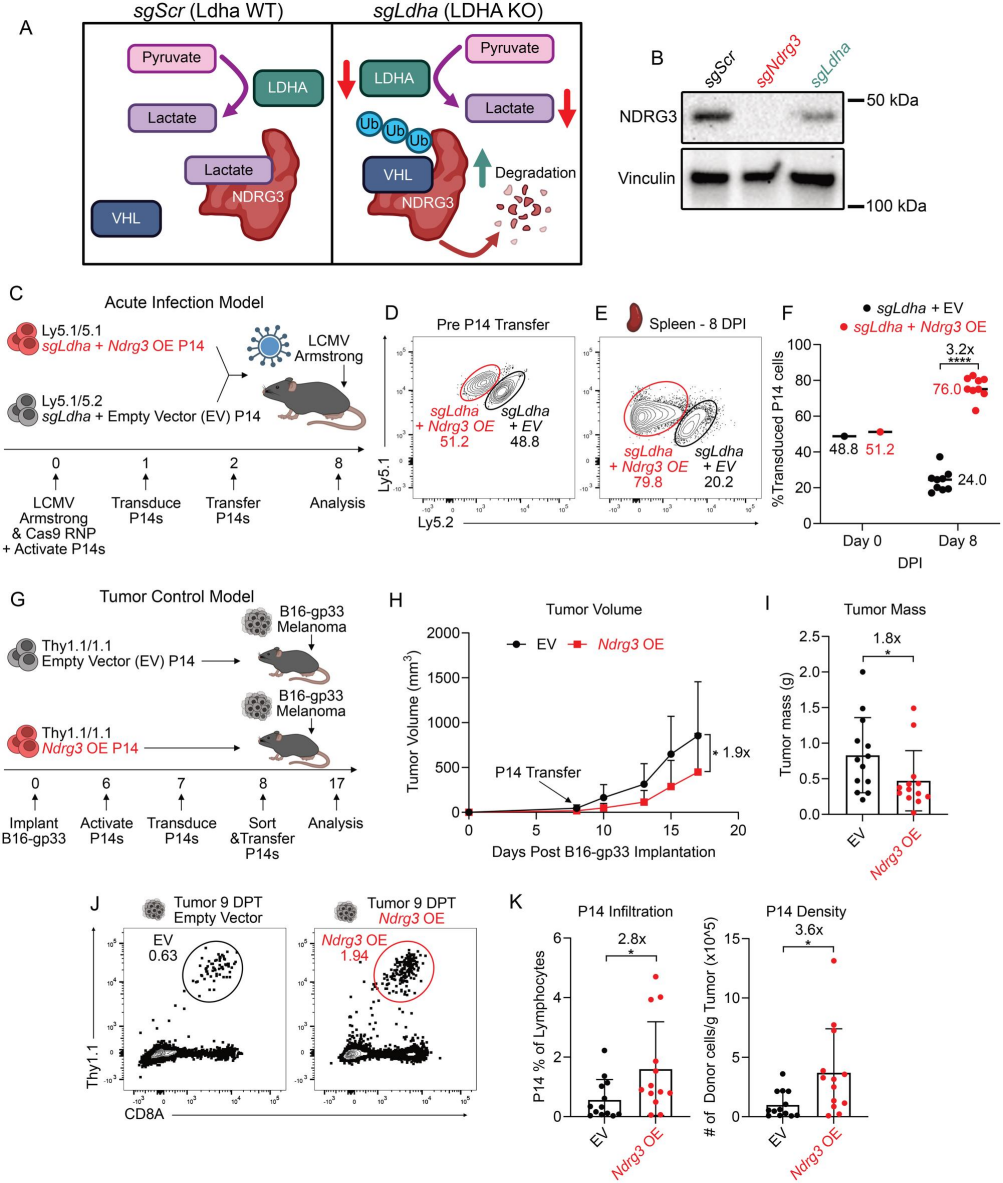
*Ndrg3* overexpression is sufficient to increase the tumor-controlling capacity of T cells. (A) Model figure displaying how the loss of lactate production through the deletion of LDHA is reported to lead to reduced NDRG3 protein levels. (B) Western blot showing NDRG3 in *sgScr*, *sgNdrg3*, and *sgLdha* T cells that have been cultured in vitro for 5 d in 10 ng/mL IL-2. (C) Experimental schematic of cotransfer experiment in which *sgLdha +* EV and *sgLdha + Ndrg3* OE P14^+^ cells with different Ly5 congenic markers were mixed at a 1:1 ratio then transferred into B6 recipient mice that were infected with LCMV-Armstrong 2 d prior. (D, E) Representative flow plots showing the frequency of *sgLdha +* EV and *sgLdha + Ndrg3* OE P14^+^ cells pretransfer (D) and in the spleen 8 DPI with LCMV-Armstrong (E). Each plot is gated on Ly5.1^+^ GFP^+^ (expressed in transduced cells) P14^+^ T cells showing Ly5.1 and Ly5.2 expression to identify the different cell populations as indicated. (F) Quantification of the frequency of *sgLdha +* EV and *sgLdha + Ndrg3* OE P14^+^ cells pretransfer and in the spleen 8 DPI with LCMV-Armstrong. Data are representative of 2 independent biological Continuedreplicates (n = 9). A 2-sided Student’s *t* test was used. Fold change was calculated using (the frequency of *sgLdha + Ndrg3* OE P14+ cells)/(the frequency of *sgLdha +* EV P14^+^ cells). (G) Experimental schematic of single genotype-transfer experiment in which EV and *Ndrg3* OE P14^+^ cells were transferred into B6 recipient mice implanted with subcutaneous B16-gp33 melanomas 8 d prior to assess tumor controlling capacity. (H) Tumor volume of B16-gp33 tumors determined by caliper measurements between 8 and 17 d post-implantation. (I) Tumor mass of B16-gp33 tumors at assay endpoint (17 d post-implantation). (J) Representative flow plots (gated on live lymphocytes) showing the frequency of EV and *Ndrg3* OE P14^+^ TILs 9 DPT (17 d post-implantation). Each plot shows Thy1.1 and CD8A expression to identify the transferred cell population. (K) Quantification of the frequency of EV and *Ndrg3* OE P14^+^ TILs 9 DPT (17 d post-implantation) within the live lymphocytes in the tumor (left). (K) Quantification of the density of EV and *Ndrg3* OE P14^+^ TILs 9 DPT (17 d post-implantation) (right). This number was determined by calculating the total number of transferred P14^+^ TILs in each tumor and dividing this number by the mass of the tumor. Data are pooled across 3 independent biological replicates (n = 13 for EV, n = 13 for *Ndrg3* OE). A 2-way ANOVA test with Šidák’s multiple comparisons correction was performed to assess comparisons between EV and *Ndrg3* OE at each time point in the tumor growth curve. A 2-sided Student’s *t* test was used to assess the differences in tumor mass, P14^+^ cell infiltration, and P14^+^ cell density. *P < 0.05, **P < 0.01, ***P < 0.001, ****P < 0.0001, ns, not significant. VHL, Von Hippel–Lindau protein; WT, wild-type.

Given that NDRG3 loss severely impairs CD8^+^ T cell expansion during infection and cancer, and NDRG3 has been reported to be stabilized by lactate (a metabolite abundant in highly glycolytic and proliferating effector T cells) (Fig. 5A),^3^^,^^35^^,^^54–58^ we asked whether lactate levels could regulate NDRG3 abundance in activated T cells. Moreover, this question was further motivated by reports showing that T cells lacking lactate dehydrogenase A (LDHA), the enzyme responsible for converting pyruvate to lactate, have severe expansion defects in response to acute viral infection,^59^^,^^60^ a phenotype that closely mirrors the effects observed in NDRG3-deficient T cells. Thus, we hypothesized that LDHA-deficient T cells might have reduced NDRG3 levels, which could contribute to their impaired expansion capacity.

To test this, we first deleted LDHA in P14^+^ T cells using CRISPR-Cas9 RNP electroporation and this demonstrated that LDHA-deficient CD8^+^ T cells had reduced NDRG3 protein by Western blot after 5 d of in vitro culture (Fig. 5B). We next tested whether overexpression of *Ndrg3* could rescue the expansion of LDHA-deficient T cells during acute LCMV-Armstrong infection. To achieve this, *sgLdha* P14^+^ cells were transduced with retroviruses overexpressing *Ndrg3* (*Ndrg3* OE) or an EV control and then these cells were cotransferred at a 1:1 ratio into LCMV-Armstrong–infected mice (Fig. 5C). We observed that the *sgLdha + Ndrg3* OE P14^+^ cells comprised 76% of the transferred sgRNA P14^+^ pool at 8DPI, reflecting a 3-fold increase relative to the *sgLdha +* EV P14^+^ cells (Fig. 5D–F) and demonstrating that *Ndrg3* OE could augment and partially rescue the expansion capacity of LDHA-deficient T cells. These findings imply that NDRG3 levels in T cells are lactate responsive and that reduced NDRG3 contributes to the impaired expansion of LDHA-deficient T cells.

Because *Ndrg3* overexpression partially rescued the expansion defect of *sgLdha* P14^+^ cells, we next assessed whether *Ndrg3* OE in wild-type cells could impart beneficial effects to T cells and improve outcomes in a clinically relevant disease model, like cancer. To examine this, equal numbers of *Ndrg3* OE P14^+^ cells and EV P14^+^ cells were transferred into separate mice bearing subcutaneous B16-gp33 melanoma flank tumors that express the gp_33-41_ epitope (Fig. 5G). Tumors in mice receiving *Ndrg3* OE P14^+^ cells were 2-fold smaller in both volume and mass (Fig. 5H, I). *Ndrg3* OE also increased P14^+^ tumor-infiltrating lymphocyte (TIL) frequency by 2.8-fold and boosted intratumoral P14^+^ T cell density by 3.6-fold (Fig. 5J, K). However, *Ndrg3* OE did not significantly alter the expression of markers of exhaustion phenotypes (e.g. PD-1 and TIM-3) or enhance cytokine production (IFN-γ, TNF-α, IL-2), which remained similar between *Ndrg3* OE and EV P14^+^ TILs (Fig. S4A–D). These results demonstrate that *Ndrg3* OE is sufficient to enhance TIL abundance to a degree that has beneficial effects on controlling tumor progression, but does not substantially alter TIL differentiation or functionality.

## Discussion

The work presented here demonstrates that NDRG3 is indispensable for the survival of T cells in contexts with repeated antigen stimulation by protecting them from RICD. To our knowledge, it is the first report to describe a role for NDRG3 in regulating antigen-specific T cell responses to viral infection and tumor challenges, uncovering a new essential regulator of activated T cell persistence and durability. This study also establishes NDRG3 as a key negative regulator of RICD and FAS signaling in T cells, adding a previously undescribed modulator to those well-characterized, vital processes for T cell homeostasis and function.

Stimulation of the TCR is requisite to activate, and sustain, the programs that empower T cells for pathogen clearance, like rapid proliferation and differentiation into effector cells.^61^^,^^62^ However, stimulation of the TCR also activates regulatory programs, like RICD, that limit the magnitude of the T cell response to avoid immunopathology.^11^ Since TCR restimulation simultaneously delivers both pro-growth signals that drive clonal expansion and pro-apoptotic cues that restrain T-cell accumulation, T cells must precisely balance these opposing inputs to successfully sustain productive responses to pathogenic stimuli. The loss of NDRG3 leads to an almost complete eradication of the CD8^+^ T cell response to tumors and viral pathogens (a 10- to 40-fold reduction in CD8^+^ T cells). Without NDRG3 present to appropriately balance the active signaling networks in the T cell, pro-apoptotic programs overpower the cell and destine it for elimination. These findings strongly suggest that NDRG3 plays an essential role in controlling the decision for T cells to undergo programmed cell death or continued survival upon repeated antigen exposure, which is paramount for T cells to successfully clear pathogenic challenges.

An important finding in this study is that the loss of NDRG3 dramatically sensitizes activated T cells to RICD, marked by enhanced cell death and CASP8 cleavage upon TCR restimulation. Given that FAS signaling activates CASP8 to achieve apoptosis and FAS activation is canonically associated with the process of RICD,^18^^,^^63^^,^^64^ our findings support the notion that *sgNdrg3* T cells are perishing due to FAS-mediated RICD. Indeed, deleting FAS in *sgNdrg3* T cells abrogates the enhanced RICD and CASP8 cleavage, indicating that NDRG3 negatively regulates FAS signaling. The exact molecular interactions that allow NDRG3 to mediate this effect are unknown. However, NDRG3 has reported roles as a scaffold protein and regulator of protein turnover,^34^^,^^35^ suggesting that it could directly interact with components of the FAS signaling pathway. One plausible model is that NDRG3 interacts with c-FLIP, a key inhibitor of FAS-mediated CASP8 activation, to facilitate c-FLIP’s recruitment to the active FAS signaling complex. In this proposed mechanism, the loss of NDRG3 would reduce c-FLIP activity and consequently enhance FAS-mediated CASP8-dependent RICD. However, FAS-mediated RICD is not solely dependent on FAS activation, as work from the Lenardo group has shown that TCR and FAS coengagement is required for maximal FAS-mediated RICD.^23^ This suggests that a molecular crosstalk exists between the two signaling pathways. Thus, given that there is more FAS-mediated RICD present in *sgNdrg3* T cells, NDRG3 could act as a negative regulator of the TCR-FAS crosstalk event and the loss of NDRG3 would then allow this pro-apoptotic signaling cascade to propagate forward more efficiently. Altogether, the observations in this study position NDRG3 as a critical negative regulator of FAS signaling during repeated antigen stimulation in T cells.

Though the primary focus of this work is on the role of NDRG3 in activated T cells, Komorowska et al.^38^ showed that the loss of NDRG3 in thymocytes had a minimal effect on thymic development, but impaired the transition from recent thymic emigrants to mature peripheral naïve T cells in the spleen. They showed that this effect could be partially rescued with the use of P14^+^ T cells and their high-avidity TCR but not with T cells that have a low-avidity TCR. Their hypothesis was that NDRG3 likely modulates an important downstream signal of TCR signaling during naïve T cell maturation given the divergence in capacity to rescue naïve T cell development between high- and low-avidity TCRs. Interestingly, our study finds that NDRG3 also plays a role in regulating a process that is dependent on TCR signaling, RICD. This suggests that both studies point toward a role for NDRG3 in modulating signaling events that are either directly or peripherally downstream of TCR stimulation. While our work highlights the profound effect of NDRG3 in regulating RICD in T cells, we also found a marginal impact of NDRG3 on naïve T cell homeostatic proliferation. Taken together with the observations from Komorowska et al., we believe these collective findings support the idea that NDRG3 exerts pleiotropic effects across multiple facets of T cell biology. Because NDRG3 has reported scaffolding capabilities, it is plausible that NDRG3’s diverse effects in T cells could be due to it having a broad set of interacting partners. For reference, NDRG1 and NDRG3 share structural similarities as they are both part of the NDRG family and NDRG1 was shown to have almost 60 interacting partners in a co-immunoprecipitation–mass spectrometry analysis.^31^^,^^65^ Given this, it opens the possibility for NDRG3 to also associate with a number of different molecular players that could be regulated in a cell state–specific manner to achieve this modulation of multiple aspects of T cell biology.

The results reported here demonstrate that T cell viability during the response to antigen-driven challenges is acutely dependent on the presence of NDRG3 protein. Notably, NDRG3 protein has been shown to be lactate-responsive in other systems,^35^ in which the absence of lactate can reduce NDRG3 levels through enhancing NDRG3 degradation. In accordance with these observations, we demonstrate that the loss of LDHA in T cells has the capacity to moderately reduce NDRG3 protein levels. It is well appreciated that glycolytic metabolism and the production of lactate are induced in T cells upon activation.^3^ Similarly, T cells also become more susceptible to RICD after activation and with the acquisition of enhanced glycolysis.^10^^,^^66^ Thus, NDRG3’s lactate responsiveness may couple a T cell’s glycolytic activity to stabilizing NDRG3 protein levels, providing a mechanism to help combat the heightened RICD that accompanies activation-induced increases in glycolysis.

The findings from this study position NDRG3 as a critical determinant of T cell fitness, raising important considerations about how modulating NDRG3 protein levels might be leveraged to reshape T cell behavior in disease contexts. Given that the loss of NDRG3 profoundly sensitizes activated T cells to RICD, targeting NDRG3 in T cell–dependent autoimmune disorders could be a useful therapeutic strategy to help eliminate self-reactive, or hyperreactive, T cells while minimally impacting the nonreactive pool. For example, patients with XLP-1 (X-linked lymphoproliferative disorder type 1) present with excessive lymphoproliferation and RICD-resistant T cells upon Epstein-Barr virus infection, leading to an almost 90% fatality rate if left untreated.^67^ However, rituximab is now used to mitigate acute symptoms of XLP-1 in order to allow patients to receive a hematopoietic stem cell transplantation, which can function as a cure. In this context, reducing NDRG3 in T cells could offer a new therapeutic modality to pair with rituximab to limit pathogenic T cell expansion in these patients, given that rituximab is not 100% effective at quelling symptoms.^68^ Additionally, we show that *Ndrg3* OE has the capacity to increase T cell infiltration into tumors, which resulted in reduced tumor burden. This demonstrates that NDRG3 could potentially augment chimeric antigen receptor T cell–based therapies for cancer and become part of the suite of genetic modulations performed on chimeric antigen receptor T cells before infusion into patients.

In summary, we unveil NDRG3 as a critical regulator of antigen-driven T cell responses and demonstrate that the loss of NDRG3 severely enhances cell death in restimulated T cells, indicating that NDRG3 normally functions as a crucial protector of T cells from RICD. Future work will be needed to understand the molecular mechanisms that endow NDRG3 with the ability to dampen RICD, including assessing NDRG3’s capacity to form physical and functional interactions with components of the FAS signaling complex, like c-FLIP, FADD, and CASP8. Finally, not only does this work identify a completely novel essential regulator of activated T cells, it also deepens our understanding of the biology of NDRG3 given that the fundamental functions and roles of this protein are still being uncovered.

## Supplementary Material

vkag070_Supplementary_Data

## Data Availability

Data for all figures are available upon request.
