# Supplementary material for: NDRG3 is essential for sustaining antigen-driven T cell responses by protecting against restimulation-induced cell death
Source: J Immunol. 2026 May 14;215(5):vkag070. doi: 10.1093/jimmun/vkag070 (PMC13176615; doi:10.1093/jimmun/vkag070)
Supplement: vkag070_Supplementary_Data [file vkag070_supplementary_data.pdf]

Figure s1: *Ndrg3* knockdown reduces prevalence of CD8⁺ T cells in LCMV-Armstrong

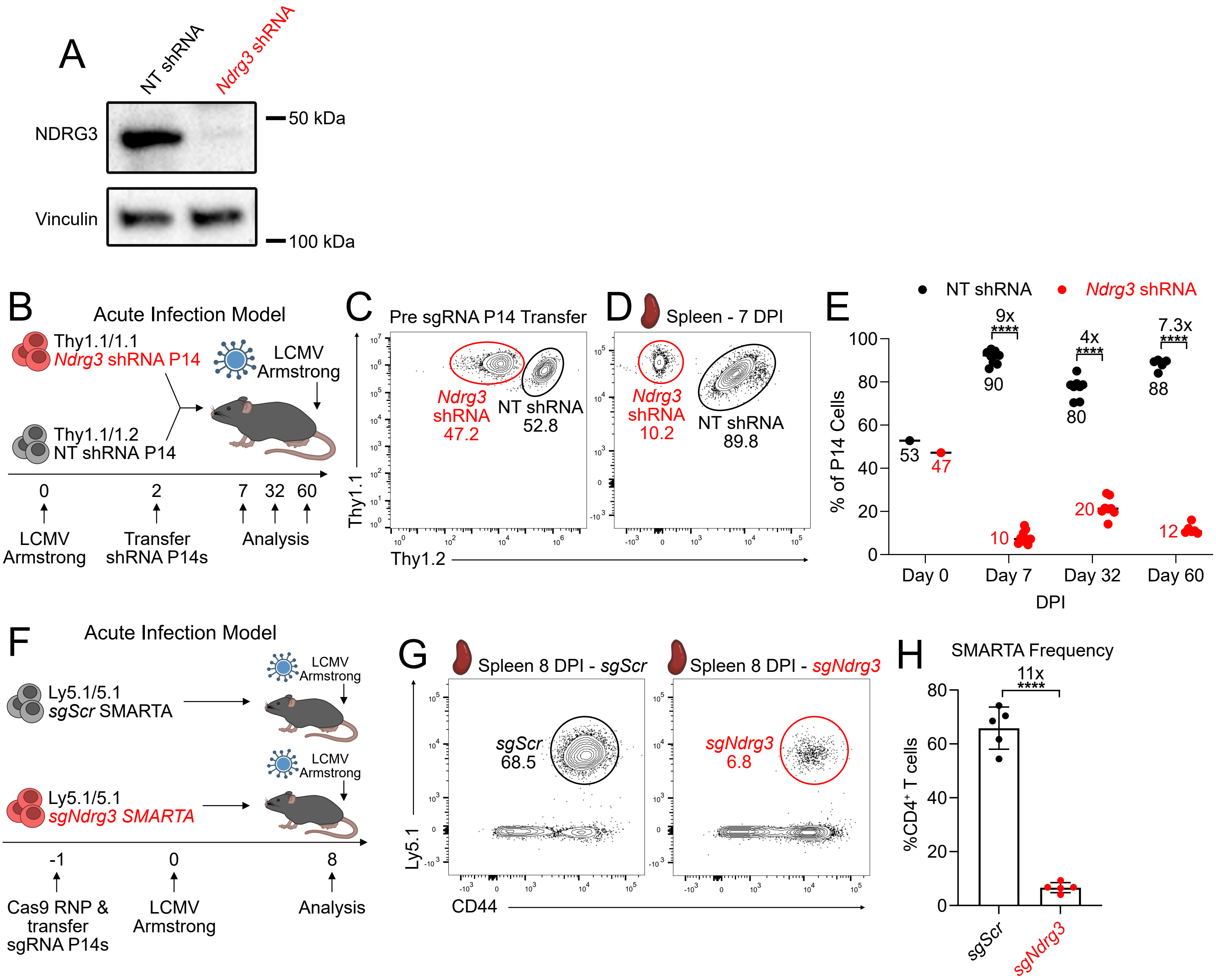

Figure s1: *Ndrg3* knockdown reduces prevalence of $CD8^+$ T cells in LCMV-Armstrong

A, Western blot showing NDRG3 levels in control Non-targeting (NT) shRNA and *Ndrg3* shRNA $P14^+$ cells that had been cultured *in vitro* for 4 days after transduction. Western blot is representative of 3 independent biological replicates. **B**, Experimental schematic of co-transfer experiment where NT shRNA and *Ndrg3* shRNA $P14^+$ cells with different Thy1 congenic markers were mixed at a 1:1 ratio then transferred into B6 recipient mice that were infected with LCMV-Armstrong two days prior. **C** & **D**, Representative flow plots showing the frequency of NT shRNA and *Ndrg3* shRNA $P14^+$ cells pre-transfer (**C**) and in the spleen 7 DPI with LCMV-Armstrong (**D**). Each plot is gated on $Ametrine^+$ (transduction indicator) $Thy1.1^+$ $P14^+$ T cells showing Thy1.1 and Thy1.2 expression to identify the different cell populations as indicated. **E**, Quantification of the frequency of NT shRNA and *Ndrg3* shRNA $P14^+$ cells pre-transfer and in the spleen at 7, 32, and 60 DPI with LCMV-Armstrong. Data are representative of 2 independent biological replicates (n = 9 for day 7, n = 8 for day 32, and n = 6 for day 60). A two-way ANOVA test with Šidák's multiple comparisons correction was performed to assess comparisons between NT shRNA and *Ndrg3* shRNA at each time point in the LCMV-Armstrong setting. Fold change was calculated using: (the frequency of NT shRNA P14+ cells) / (the frequency of *Ndrg3* shRNA $P14^+$ cells). **F**, Experimental schematic of single genotype-transfer experiment where *sgScr* and *sgNdrg3* $SMARTA^+$ cells were transferred into B6 recipient mice that were subsequently infected with LCMV-Armstrong one day later. **G**, Representative flow plots showing the frequency of *sgScr* (left panel) and *sgNdrg3* $SMARTA^+$ cells (right panel) in the spleen at 8 DPI with LCMV-Armstrong. Each plot is gated on $CD4^+$ T cells showing Ly5.1 and CD44 expression to identify the transferred cell population. **H**, Quantification of the frequency of *sgScr* and *sgNdrg3* $SMARTA^+$ cells in the spleen at 8 DPI with LCMV-Armstrong. Data are representative of 2 independent biological replicates. A two-sided

Student's t-test was used in the SMARTA^{+} sgRNA setting. *P < 0.05, **P < 0.01, ***P < 0.001, ****P < 0.0001

Figure s2: Cytokine withdrawal does not selectively reduce *sgNdrg3* P14 cell frequency

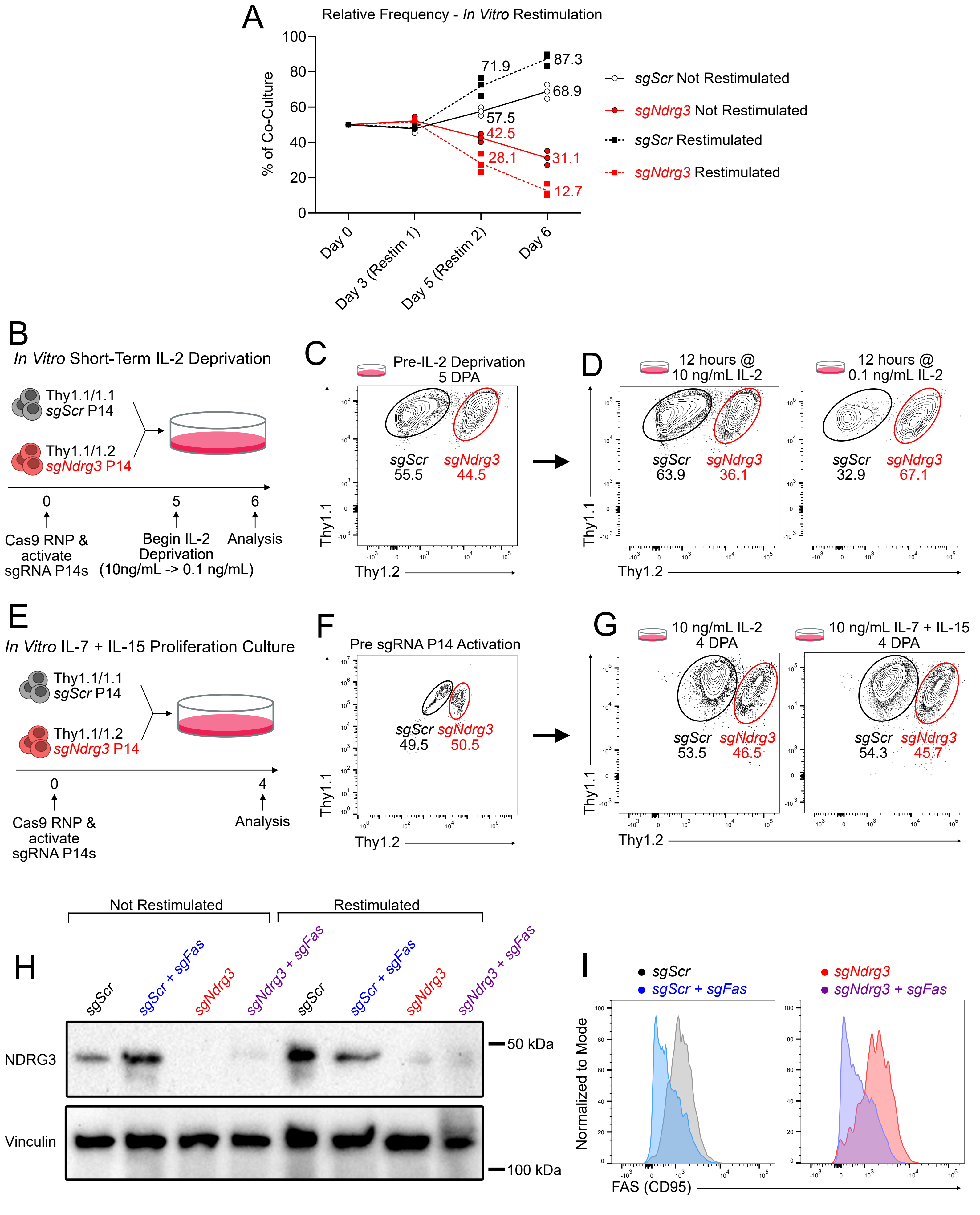

Figure s2: Cytokine withdrawal does not selectively reduce *sgNdrg3* P14 cell frequency

A, Quantification of the frequency of *sgScr* and *sgNdrg3* $P14^+$ cells pre-activation and at 3, 5, and 6 DPA in the co-culture restimulation assay. Data are pooled across 3 independent biological replicates. **B**, Experimental schematic of co-culture experiment where *sgScr* and *sgNdrg3* $P14^+$ cells with different Thy1 congenic markers were mixed at a 1:1 ratio, activated with plate-bound anti-CD3 and anti-CD28, and then cultured in 10 ng/mL IL-2 *in vitro* for 5 days before beginning a 12 hour IL-2 deprivation. The co-culture was split into 2 conditions with one receiving the standard 10 ng/mL IL-2 for 12 hours and one receiving 0.1 ng/mL IL-2 for 12 hours. **C** & **D**, Representative flow plots showing the frequency of *sgScr* and *sgNdrg3* $P14^+$ cells pre-IL-2 deprivation (**C**) and 12 hours post IL-2 deprivation (**D**). **D**, The left panel shows the co-culture condition that received 10 ng/mL IL-2 and the right panel shows the co-culture condition that received 0.1 ng/mL IL-2. Each plot is gated on $Thy1.1^+$ $P14^+$ T cells showing Thy1.1 and Thy1.2 expression to identify the different cell populations as indicated. Data are representative of 2 independent biological replicates. **E**, Experimental schematic of co-culture experiment where *sgScr* and *sgNdrg3* $P14^+$ cells with different Thy1 congenic markers were mixed at a 1:1 ratio, activated with plate-bound anti-CD3 and anti-CD28, and then cultured in 10 ng/mL IL-7 & 10 ng/mL IL-15 *in vitro* for 4 days. **F** & **G**, Representative flow plots showing the frequency of *sgScr* and *sgNdrg3* $P14^+$ cells pre-activation (**F**) and 4 DPA *in vitro* (**G**). **G**, The left panel shows the co-culture condition that received 10 ng/mL IL-2 and the right panel shows the co-culture condition that received 10 ng/mL IL-7 & 10 ng/mL IL-15. Each plot is gated on $Thy1.1^+$ $P14^+$ T cells showing Thy1.1 and Thy1.2 expression to identify the different cell populations as indicated. Data are representative of 2 independent biological replicates. **H**, Western blot showing NDRG3 levels in all genotypes (*sgScr*, *sgScr* + *sgFas*, *sgNdrg3*, and *sgNdrg3* + *sgFas*) and restimulation conditions

(not restimulated and restimulated) 6 DPA. Western blot is representative of 3 independent biological replicates. **I**, FAS (CD95) staining in *sgScr*, *sgScr* + *sgFas*, *sgNdrg3*, and *sgNdrg3* + *sgFas* 6 DPA in the restimulation assay setting. FAS staining is representative of 3 independent biological replicates.

Figure s3: NDRG3 does not modulate the abundance of cell viability regulators

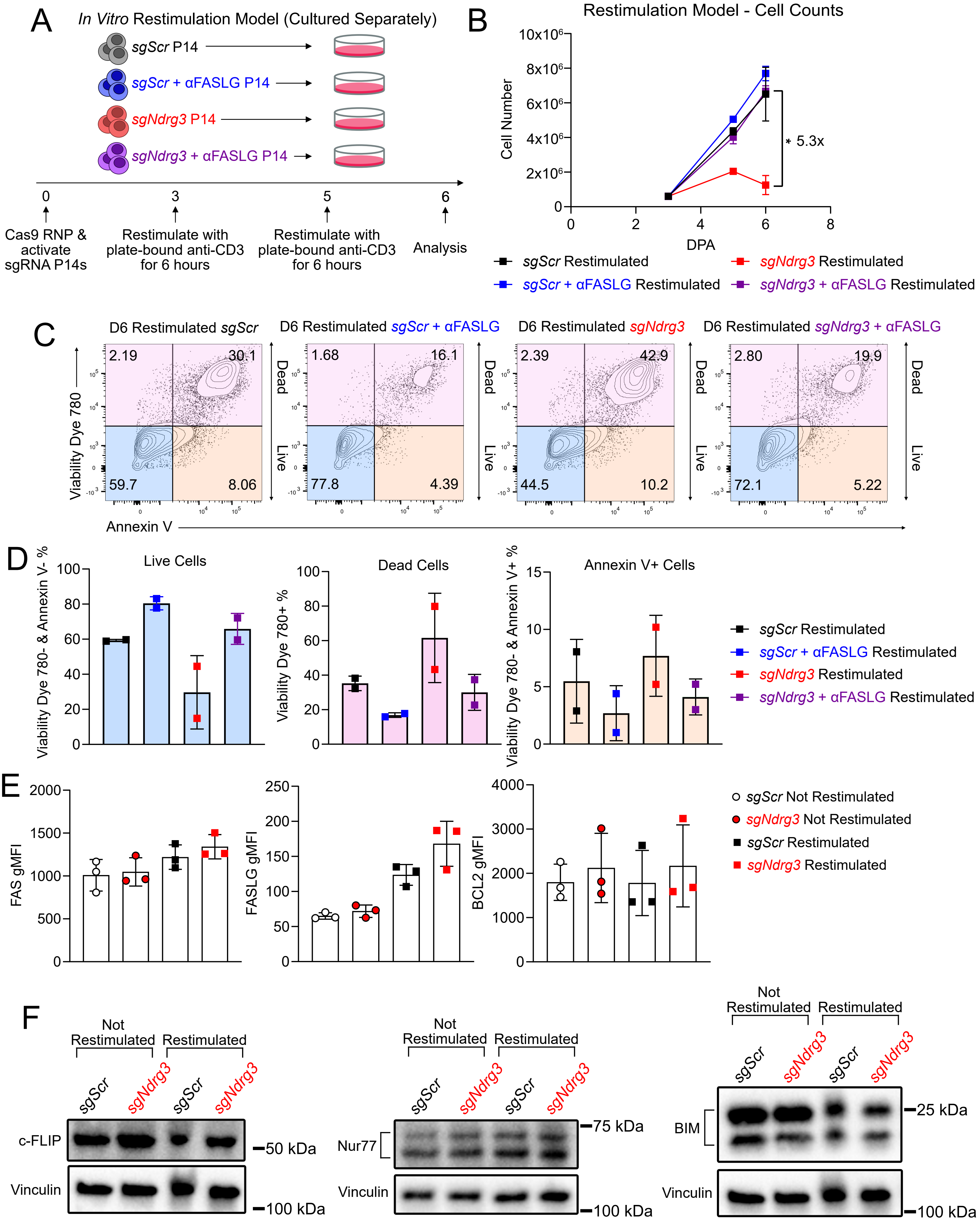

Figure s3: NDRG3 does not modulate the abundance of cell viability regulators

A, Experimental schematic of *in vitro* restimulation assay where the experimental groups were cultured separately including *sgScr*, *sgScr* + αFASLG*, sgNdrg3,* and *sgNdrg3* + αFASLG P14$^+$ cells. FASLG blocking antibody treatment (7.5 μg/mL) was started at 3 DPA at the beginning of the first restimulation and continued until the end of the assay. **B**, Quantification of the number of cells present in the *in vitro* restimulation assay at 3, 5, and 6 DPA. Data are pooled across 2 independent biological replicates. **C & D**, Cells from the restimulation assay were stained at 6 DPA with Viability Dye 780 and Annexin V to identify live cells (Viability Dye 780$^-$ & Annexin V$^-$), dead cells (Viability Dye 780$^+$), and Annexin V$^+$ cells (Viability Dye 780$^-$ & Annexin V$^+$) as shown in representative flow plots **(C)** and quantified in bar graphs **(D)**. Data are pooled across 2 independent biological replicates. **E**, Quantification of the geometric mean fluorescence intensity (gMFI) of FAS, FASLG, and BCL2 in *sgScr* and *sgNdrg3* P14$^+$ cells at 6 DPA. Data are pooled across 3 independent biological replicates. **F**, Western blots showing c-FLIP, Nur77, BIM, and Vinculin at 6 DPA. Western blots are representative of 3 independent experiments. A two-way ANOVA with Šidák's multiple comparisons correction was performed to assess each comparison in the single-genotype culture restimulation assay. A one-way ANOVA with Tukey's multiple comparisons correction was used to assess the bar graphs showing the proportion of live, dead, and Annexin V$^+$ cells as well as the expression of FAS, FASLG, and BCL2. *$P < 0.05$, **$P < 0.01$, ***$P < 0.001$, ****$P < 0.0001$

Figure s4: *Ndrg3* overexpression minimally alters T cell exhaustion differentiation during the anti-tumor response

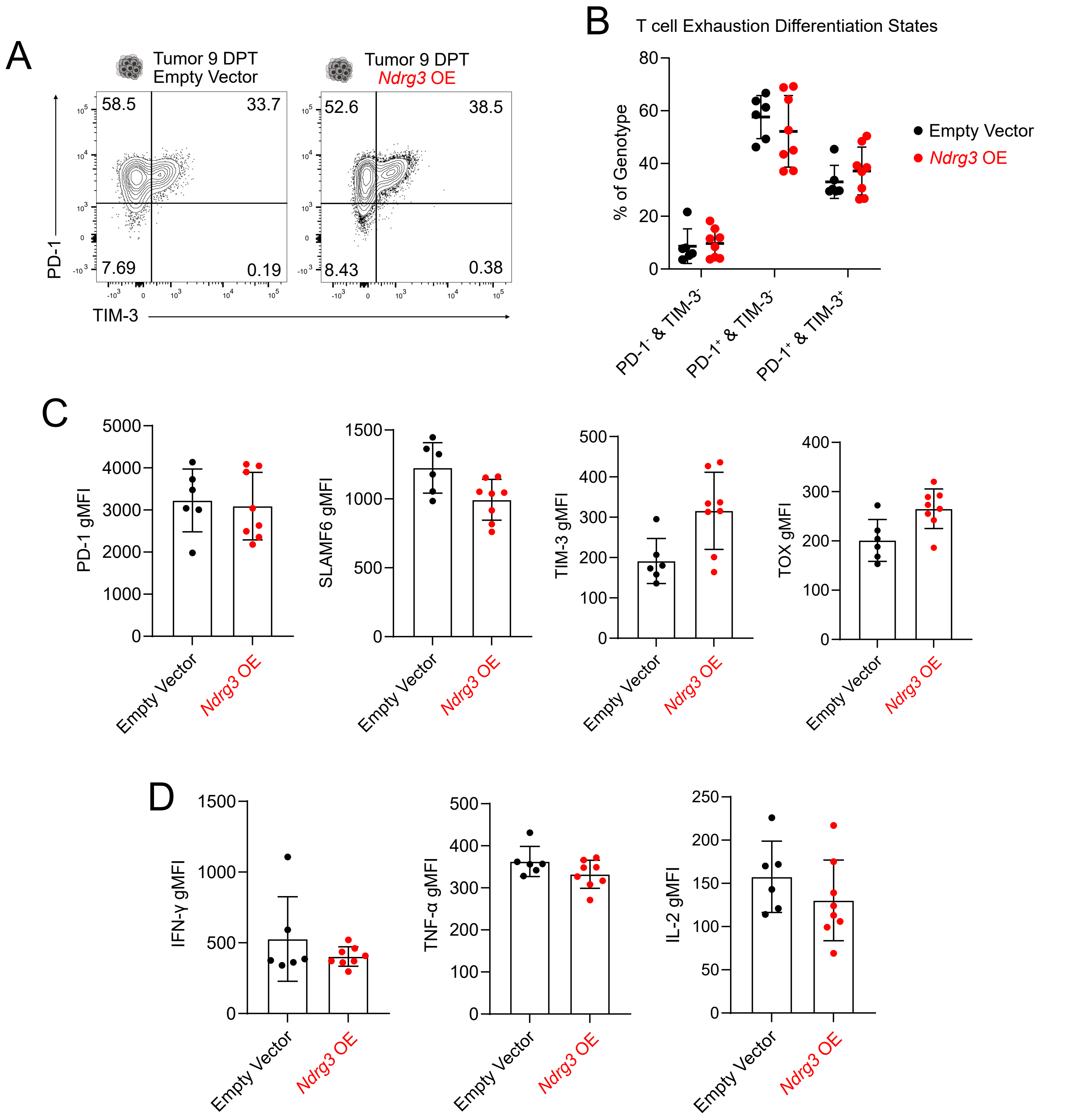

Figure s4: *Ndrg3* overexpression minimally alters T cell exhaustion differentiation during the anti-tumor response

A, Representative flow plots showing PD-1 and TIM-3 staining on Empty Vector (EV) and *Ndrg3* OE P14$^+$ TILs 9 DPT. The left panel shows the EV P14$^+$ TILs and the right panel shows the *Ndrg3* OE P14$^+$ TILs. Each plot is gated on Thy1.1$^+$ P14$^+$ T cells. **B**, Quantification of the frequency of PD-1$^-$ & TIM-3$^-$, PD-1$^+$ & TIM-3$^-$, and PD-1$^+$ & TIM-3$^+$ subtypes of T cell exhaustion for both EV and *Ndrg3* OE P14$^+$ TILs 9 DPT. **C**, Quantification of the gMFI of PD-1, SLAMF6, TIM-3, and TOX in both EV and *Ndrg3* OE P14$^+$ TILs 9 DPT. **D**, Quantification of the gMFI of IFN-γ, TNF-α, and IL-2 in both EV and *Ndrg3* OE P14$^+$ TILs 9 DPT. Cells were stimulated in T cell media in the presence of ionomycin, PMA, and brefeldin A for 5 hours. Data are representative of 3 independent biological replicates (n = 6 for EV P14$^+$ TILs and n = 8 for *Ndrg3* OE P14$^+$ TILs).
